# Elucidation of anti-SARS-CoV-2 and anti-inflammatory bioactives in Qingyan Dropping Pills via integrated *in silico* screening and bioactivity validation

**DOI:** 10.3389/fmed.2025.1684713

**Published:** 2025-11-10

**Authors:** Liting Liu, Xinru Li, Xinyue Wang, Peng Zhang, Qi Yang, Tong Geng, Yuefei Wang, Junhua Zhang, Changjian Wang, Jing Yang, Min Zhang

**Affiliations:** 1 State Key Laboratory of Chinese Medicine Modernization, Tianjin University of Traditional Chinese Medicine, Tianjin, China; 2 Guangzhou Laboratory, Guangzhou, China; 3 Tianjin Pharmaceutical Da Ren Tang Group Corp., Ltd. Traditional Chinese Medicine Research Institute, Tianjin, China; 4 Haihe Laboratory of Modern Chinese Medicine, Tianjin, China

**Keywords:** COVID-19, SARS-CoV-2, Qingyan Dropping Pills (QDP), antivirus, anti-inflammation

## Abstract

**Background:**

The global outbreak of coronavirus disease (COVID-19), caused by severe acute respiratory syndrome coronavirus 2 (SARS-CoV-2), has raised significant public health concerns. Qingyan Dropping Pills (QDP), as a recommended drug, is issued by the National Health Commission of the People’s Republic of China for the treatment of COVID-19. However, its bioactive compounds and their mechanisms of action remain largely unidentified. In this study, the integration of computational and experimental approaches was performed to identify the bioactive compounds in QDP and elucidate its mechanisms against COVID-19.

**Methods:**

Utilizing UPLC-Q/TOF-MS, the chemical compounds of QDP were delineated, followed by network pharmacology analysis and molecular docking targeting SARS-CoV-2 spike protein (S^pro^), main protease (M^pro^), and papain-like protease (PL^pro^). To validate the inhibitory activity of these compounds, fluorescence resonance energy transfer (FRET) and surface plasmon resonance (SPR) assays were employed. The antivival efficacy was tested in Vero E6 cells infected with SARS-CoV-2 Omicron BA.5 variant. Moreover, anti-inflammatory potential was evaluated via the measurement of inflammatory markers, including nitric oxide (NO), interleukin-6 (IL-6), interleukin-1 beta (IL-1*β*), and tumor necrosis factor-alpha (TNF-*α*).

**Results:**

Among the 48 identified compounds, 33 demonstrated potential antiviral activity against COVID-19. Notably, Hamamelitannin (HAM), corilagin (COR), and rhoifolin (RHO) effectively interacted with S^pro^, M^pro^ and PL^pro^*in silico*. In SPR assays, the equilibrium dissociation constant (*K_D_*) for COR and RHO ranged from 4.515 × 10^−8^ M to 7.718 × 10^−6^ M, while HAM showed strong binding affinity to S^pro^ (*K_D_* = 9.33 × 10^−8^ M) but weaker affinity for M^pro^ and PL^pro^. In FRET assays, COR and RHO inhibited M^pro^ with IC_50_ valuse of 0.73 μM and 21.61 μM, respectively. Additionally, COR proved effective against the Omicron BA.5 variant. The compounds COR, HAM, RHO, isoliquiritin (ISO), glycocholic acid (GLYCH), and gallic acid (GAL) displayed significant anti-inflammatory activity by inhibiting the crucial inflammatory factors, indicating their dual therapeutic potential in managing COVID-19.

**Conclusion:**

Our study focused on Chinese patent medicine QDP to highlight the anti-SARS-CoV-2 and anti-inflammatory bioactives, providing evidence and insights into its clinical practice in the treatment of COVID-19.

## Graphical abstract

**Figure:**
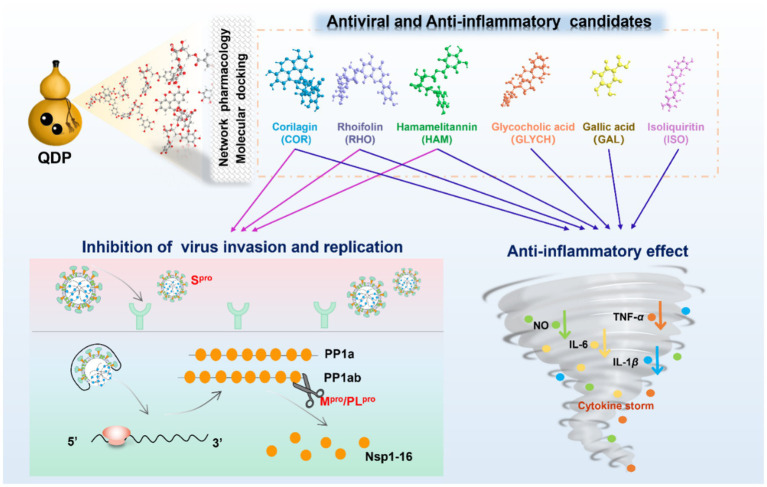

## 1 Introduction

The outbreak of COVID-19 has posed a severe threat to global public health (1). SARS-CoV-2 is the primary pathogen responsible for this pandemic. Although remarkable progress has been made in vaccine development and antiviral drug discovery-including BNT162b2, mRNA-1273, ChAdOx1, and BBIBP-CorV, as well as in therapeutic options such as remdesivir and corticosteroids, the ongoing mutation of SARS-CoV-2 and the emergence of novel variants have sustained the pandemic’s worldwide impact (1). The development of effective inhibitors against SARS-CoV-2 remains crucial in controlling viral infection. In addition, the coexistence of COVID-19 with chronic diseases, such as diabetes mellitus, has increased disease severity and mortality, highlighting the need for comprehensive prevention and treatment strategies. Traditional Chinese medicine (TCM) has played a significant role in the fight against COVID-19, owing to its holistic regulatory effects, multi-target therapeutic mechanisms, and personalized treatment approaches (2–4). Among the various treatments used in clinical practice, three drugs-Jinhua Qinggan granule, Lianhua Qingwen capsule, and Xuebijing injection-and three prescriptions-Xuanfei Baidu formula, Huashi Baidu formula, and Qingfei Paidu decoction-have demonstrated effectiveness in reducing mortality rates, alleviating symptoms, preventing the progression of mild to severe disease and promoting recovery (5, 6). These findings provide valuable insights and complementary approaches to combating emerging infectious diseases. Therefore, elucidating the bioactive compounds and underlying mechanisms is imperative for optimizing the use of TCM in clinical practice.

QDP is a multi-herbal Chinese prescription, mainly used for treating acute or chronic throat inflammation in the clinic. Based on its demonstrated efficacy, QDP has been endorsed by the National Health Commission of the People’s Republic of China as a recommended treatment for COVID-19 patients (7). The formulation comprises *Terminalia chebula* Retz. (Chebulae Fructus, CF), *Bovis Calculus Artificialis* (Artificial Calculus Bovis, ACB), Indigo Naturalis (Natural Indigo, NI), *Glycyrrhizae* Radix et Rhizoma (Licorice root, LR), Borneolum Syntheticum (borneol, BO), and Mentholum (L-menthol, LM). Previous studies have identified QDP as a common remedy for treating respiratory diseases, demonstrating inhibitory effects against pathogens such as *Staphylococcus aureus* (*S. aureus*) and *Escherichia coli* (*E. coli*) (8). Therefore, investigating the bioactive compounds and mechanisms of action that underlie its antiviral and anti-inflammatory activities is both urgent and meaningful for clinical application in managing COVID-19.

SARS-CoV-2, a single-stranded RNA virus belonging to the *β*-coronavirus family, encodes 29 proteins, including four structural proteins, sixteen non-structural proteins, and nine accessory proteins (9, 10). The life cycle of SARS-CoV-2 contains four main stages, invasion, replication, assembly, and release. S^pro^ serves as the primary target as it binds to angiotensin-converting enzyme 2 (ACE2) receptors, facilitating membrane fusion and mediating viral entry into host cells (11). Upon release of the viral genome into the host cell, viral replication is activated. M^pro^ and PL^pro^ play pivotal roles by cleaving the PP1a and PP1ab polyproteins, producing non-structural proteins essential for SARS-CoV-2 transcription and replication. Following the assembly of mature viral particles, these are released into the extracellular space via exocytosis, triggering an inflammatory response that can lead to a cytokine storm (12). Given their critical functions in the viral life cycle, S^pro^, M^pro^, and PL^pro^ are targeted for screening potential antiviral compounds (13, 14).

Accordingly, in this study, we first clarified the chemical profile of QDP using UPLC-Q/TOF-MS analysis. Subsequently, we constructed pharmacological networks to identify potential active compounds, which were filtered through molecular docking techniques. Finally, focusing on the identified compounds, SPR assay, FRET screening, antiviral experiments, and cell models were performed to validate the active compounds and elucidate their mechanisms of the antivirus and anti-inflammation. This study aims to contribute valuable insights and robust scientific foundation into the clinical application of QDP for treating COVID-19 patients.

## 2 Materials and methods

### 2.1 Reagents and materials

The QDP (Lot. 620,054) were generously provided by Tianjin pharmaceutical DA REN TANG group corporation limited NO.6 traditional Chinese medicine factory (Tianjin, China). High-quality MS grade methanol and acetonitrile were purchased from Thermo Fisher Scientific Co., Ltd. (Fair Lawn, NJ, USA), as well as formic acid and dimethyl-sulfoxide (DMSO) from Shanghai Aladdin Biochemical Technology Co., Ltd. (Shanghai, China). M^pro^ and PL^pro^ were purified in our lab and S^pro^ was purchased from Sino Biological Inc. (Beijing, China). CM5 sensor chips, amine-coupling kit, and running buffer were purchased from General Electric Company (Boston, USA). Dulbecco’s modified Eagle’s medium (DMEM) and fetal bovine serum were purchased from Gibco Invitrogen Corp. (New York, USA). Nitric oxide (NO) assay kit was purchased from the Beyotime Institute of Biotechnology (Shanghai, China). Enzyme-linked immunosorbent assays (ELISA) kits of IL-6, TNF-*α*, and IL-1*β* were supplied by ZCIBIO Technology Co., Ltd. (Shanghai, China).

The 24 standard compounds (HPLC purity ≥ 98%) were used as the reference standards. 24 compounds are derived from the three institutions, National Institutes for Food and Drug Control, Sichuan Weiqi Technology Co., Ltd. and Shanghai Yuanye Bio-Technology Co., Ltd. Glycyrrhizic acid (GLYCZ), glycyrrhetinic acid (GLYCT), liquiritigenin (LIQN), liquiritin (LIQ), isoliquiritin (ISO), gallic acid (GAL), glycocholic acid (GLYCH), indirubin (IND), cholic acid (CHO), and hyodeoxycholic acid (HYO) were obtained from National Institutes for Food and Drug Control (Beijing, China) and Sichuan Weiqi Technology Co., Ltd. (Sichuan, China). Rhoifolin (RHO), corilagin (COR), hamamelitannin (HAM), chebulic acid (CHE), tauroursodeoxycholic acid (TAUD), erucamide (ERU), licoricesaponin G2 (LIC), ononin (ONO), glycoursodeoxycholic acid (GLYCA), piperine (PIP), glycycoumarin (GLYC), 16-hydroxyhexadecanoic acid (HYDRA), chenodeoxycholic acid (CHEA) and oleamide (OLE) were purchased from Shanghai Yuanye Bio-Technology Co., Ltd. (Shanghai, China). Ritonavir (RIT) as a positive drug was obtained from Shanghai Yuanye Bio-Technology Co., Ltd. (Shanghai, China).

### 2.2 Preparation of reference and sample solutions for chemical analysis

QDP (80 mg) was extracted with 3 mL 70% methanol (*v/v*) by ultrasound extraction for 30 min at 35 °C, filtered through a 0.45 μm nylon syringe filter. The filtrate was collected for further analysis.

The reference compounds were dissolved in methanol with the concentration of 1 mg/mL as the standard stock solutions, respectively. Then, the reference stock solutions were mixed and diluted with methanol to obtain the standard working solution. The final concentration for each compound was 20 μg/mL.

### 2.3 UPLC-Q/TOF-MS analysis

Analysis was performed on an Agilent 1,290 coupled to an Agilent 6,520 Q/TOF MS spectrometer with an electrospray ionization (ESI) source (CA, USA), which was operated by a MassHunter workstation. Gradient elution was undertaken on the ACQUITY UPLC® BEH C18 (2.1 × 100 mm, 1.7 μm) at 35 °C. The mobile phase consisted of acetonitrile (A) and water with 0.1% formic acid (B), which was applied as follows: 0–4 min, 5% − 9% A; 4–10 min, 9% − 44.5% A; 10–12 min, 44.5% − 51% A; 12–14 min, 51% − 58% A; 14–16 min, 58% − 62% A; and 16–25 min, 62% − 95% A. The flow rate was set at 0.3 mL/min, and the sample injection volume was 3 μL.

Mass spectra were analyzed in both positive ion and negative ion modes with a mass range of *m/z* 100–1,500 Da. Optimal MS parameters were as follows: capillary voltage, −2.5 kV in negative ion mode and +3.0 kV in positive ion mode, capilary temperature at 120 °C, desolvation temperature at 350 °C, flow rate of collision gas at 0.20 mL/min, nebulizer gas pressure at 30 psig, and collision energy at 30 eV. Data acquisition was performed using MassHunter workstation.

### 2.4 Network pharmacology analysis

Potential targets associated with the identified compounds in QDP were retrieved from TCMSP database^1^ and the Swiss Target Prediction database^2^ (15). We selected “COVID-19” and “coronavirus pneumonia” as the keywords to acquire the disease-related targets from the GeneCards database^3^ and OMIM database.^4^ Then, the shared targets were displayed in Venn diagram (16). Based on the shared targets, the herbs-preparation-compounds-targets network and protein–protein interaction (PPI) network were, respectively, constructed to screen the potential active compounds and the core targets. *Homo sapiens* was used to restrict the organism, and the minimum interaction threshold was set at the highest confidence (0.900). In the network, the degree value of the target was calculated to assess the significance (17).

Gene ontology (GO) and Kyoto Encyclopedia of Genes and Genomes (KEGG) pathway enrichment analysis were performed using the DAVID database^5^ to illustrate the biological process (BP), cellular component (CC), molecular function (MF), and signaling pathways (18). The top 10 GO terms and 30 KEGG pathways ordered by *p*-value were exhibited in the bar graphs.

### 2.5 Molecular docking

Molecular docking is a useful approach in structure-based drug discovery *in silico* (19). To further discover the bioactive compounds in QDP against SARS-CoV-2, the compounds that were filtered by network pharmacology were docked with the key targets of SARS-CoV-2, including M^pro^, PL^pro^, and S^pro^ (PDB ID: 6 LU7, 7CJM, and 7T9L). Structures of the tested compounds and protein-ligand complexes were obtained from the PubChem database^6^ and the protein data bank (PDB) database^7^ (20).

The preparation of ligands and proteins was performed using Discovery Studio 2020 software. Proteins were prepared via removing extra water molecules, adding hydrogens, and repairing missing residues. The location of the original ligand was defined as the active site, which was deleted from the prepared protein. The cdocker mode was employed for docking. Molecular docking was carried out using the cdocker protocol as implemented in Discovery Studio 2020, adhering to its validated default settings. The CHARMm-based scoring function accounted for van der Waals and electrostatic interactions via the Lennard-Jones potential and a distance-dependent dielectric constant (cut-off: 14 Å). The docking procedure involved random rigid-body rotations of the ligands, simulated annealing, and a final grid-based refinement. Pose ranking was based on the CDOCKER Interaction Energy, which comprises the ligand’s internal strain energy and its interaction energy with the protein. The results were visualized by Pymol 2.6 software. Ritonavir (RIT) as a positive compound that was reported to inhibit SARS-CoV-2 (21), N3 as the original ligand of M^pro^, and GRL0617 as the original ligand of PL^pro^, were docked to the corresponding targets as the positive references (22, 23).

### 2.6 Cloning, protein expression, and purification of SARS-CoV-2 M^pro^ and PL^pro^

The plasmid containing full-length gene of SARS-CoV-2 M^pro^ and PL^pro^ were constructed and transferred into *E. coli* BL21 (DE3), respectively, which were separately cultured in Luria broth medium with 100 μg/mL ampicillin for expressing M^pro^ and 50 μg/mL kanamycin for expressing PL^pro^ at 37 °C for 6 ~ 8 h. Then, the proteins were induced for expression with 500 μM isopropyl *β*-D-thiogalactoside (IPTG) at 16 °C for 16 ~ 20 h. A centrifugated deposit were collected by 4,000 rpm for 20 min at 4 °C, which were crushed through the high-pressure homogenizer. Ni-NTA affinity column was used for purification with the buffer at pH 8.0 containing 20 mM Tris–HCl, 150 mM NaCl, 5% Glycerol, and 300 mM imidazole. The C-terminal 6 x His tag of M^pro^ was removed by human rhinovirus 3C protease, and the small ubiquitin-like modifier (SUMO) linking to PL^pro^ was removed by SUMO enzyme. The M^pro^ and PL^pro^ were further subjected to purification by size-exclusion chromatography (Superdex 200 Increase 10/300 GL) and ion exchange chromatography (HiTrapTM Q HP 5 mL). SDS-PAGE is employed to verify the purification of the tested proteins above 95%.

### 2.7 SPR assay

Surface plasmon resonance (SPR) assay was performed by Biacore T200 to validate the binding affinity of the focused compounds to the targeted proteins. The S^pro^, M^pro^, and PL^pro^ as the target proteins were, respectively, immobilized on the activated CM5 sensor via an amine coupling reaction. Firstly, the focused compounds were dissolved with DMSO at 2.5 mM as the standard stock solution, which was diluted for 20 times with the buffer (1.05 × PBS-P solution) to obtain the initial concentration samples (125 μM). Then, the samples were diluted step by step in the running buffer (1.05 × PBS-P, 5% DMSO, pH = 7.4) with the concentration ranging from 0.030 to 125 μM. The flow rate was set at 30 μL/min, and the association time and dissociation time was separately set at 120 s and 180 s. The entire experiment was conducted at a temperature of 25 °C. Data were analyzed by Biaevaluation software 2.0 in kinetics mode.

### 2.8 Determination of the enzymatic inhibition activities on M^pro^ and PL^pro^

Fluorescence resonance energy transfer assay (FRET) was employed to screen enzymatic inhibitors according to the properties of M^pro^ and PL^pro^ as the drug targets. The substrates can specifically recognize the proteins to change the distance between the fluorescent receptor and the donor, which has been used to investigate intermolecular interactions. The substrate of M^pro^ is designed and synthesized as MCA-AVLQSGFR-Lys (Dnp)-Lys-NH_2_ and the substrate sequence of PL^pro^ is Dabcyl-FTLKGGYAPTKVTE-Edans by GL biochem Co., Ltd. (Shanghai, China). The enzymatic activity evaluating system of M^pro^ contains 0.2 μM protein, the different concentrations of compounds (0.3125–160 μM), and 20 μM of substrate. And the enzymatic activity evaluating system of PL^pro^ contains 1 μM protein, the different concentrations of compounds (0.049–100 μM), and 10 μM of substrate. The inhibitions (%) of the tested compounds were evaluated by Graphpad prism 8.0 (24).

### 2.9 Antiviral activity assay

The antiviral activity of the tested compound was evaluated by cytopathic effects (CPE) inhibition assay in Vero E6 cells *in vitro*, which was performed in a biosafety level 3 (BSL-3) laboratory. Vero E6 cells were seeded in a 96-well plate with 1 × 10^5^ cells/well overnight, which were cultured in DMEM supplemented with 10% fetal bovine serum, 100 μg/mL streptomycin, and 100 U/mL penicillin, at 37 °C with 5% CO_2_ and 95% air. The mixed sample with Omicron BA.5 (MOI = 0.01) and the tested compound (concentration from 0.0137 μM to 30.00 μM) was added into each well to incubate for 48 h at 37 °C. Then, CPE rate was scored by the Celigo Image Cytometer, which was employed to calculate the value of half maximal effective concentration (EC_50_).

### 2.10 Anti-inflammatory activity assay

RAW 264.7 cells were cultured in DMEM with 10% fetal bovine serum, 100 μg/mL streptomycin, and 100 U/mL penicillin at 37 °C and supplemented with 5% CO_2_ and 95% air. After seeded and cultured in a 96-well plate (2 × 10^5^ cells/well, 100 μL medium/well) for overnight, RAW 264.7 were treated with the tested compounds for 24 h, respectively. Then, 50 μL MTT (2.5 mg/mL) was added into each well and incubated at 37 °C for 4 h. Finally, the absorbance was recorded at 490 nm by a microplate reader to assess the cell viability.

NO is an essential pro-inflammatory factor, which can be not only highly expressed in cells but also promoted the expression of other inflammatory cytokines. TNF-*α*, IL-6, and IL-1*β* were considered as the main inflammatory cytokines, which were screened as the key targets in network pharmacology. So, the cytokines, including NO, TNF-*α*, IL-6, and IL-1*β*, were selected as indicators to evaluate the anti-inflammatory effects. After cultured in a 96-well plate overnight for 1 × 10^5^ cells/well, RAW 264.7 cells were simultaneously treated with the tested compounds (concentration from 5 μM to 100 μM) and lipopolysaccharide (LPS) (concentration with 1 μg/mL) for 24 h. Then, the supernatant was collected to measure the cytokines levels with the corresponding assay kits, respectively.

### 2.11 Statistical analysis

All statistical analyses were performed using GraphPad Prism 8. Statistical significance for each endpoint was assessed with an unpaired two-tailed Student’s *t*-test. Data are presented as the mean ± standard deviation (SD). A significant level of *p* < 0.05 was considered statistically significant for all analyses.

## 3 Results

### 3.1 Characterization of the chemical compounds in QDP

**Figure 1:**
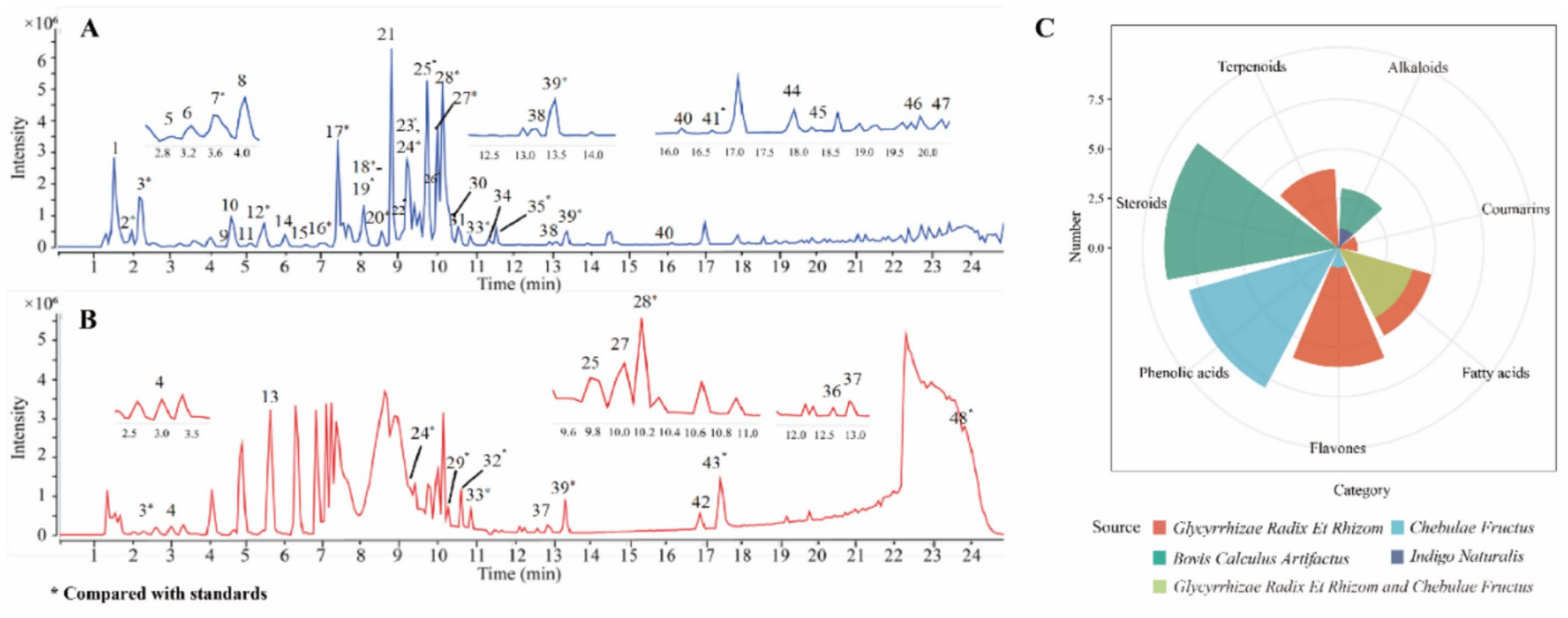
Base peak intensity chromatograms and compound source analysis of QDP extracts. **(A)** Negative ion mode. **(B)** Positive ion mode. **(C)** Radar map of compound sources.

To qualitative analysis the chemical compounds in QDP, the chromatographic analysis was performed by UPLC-Q/TOF-MS and the base peak chromatogram is shown in Figures 1A,B, from which 48 compounds (Supplementary Table S1) were tentatively identified and 24 compounds were unambiguously characterized by matching with reference compounds (GAL, LIQ, ISO, LIQN, GLYCZ, CHO, HYO, IND, GLYCT, CHE, RHO, COR, GLYCH, HAM, TAUD, ERU, LIC, PIP, OLE, GLYCA, GLYC, HYDRA, HYO, and CHEA). The radar map summarizes the cross-herb distribution of major chemical classes. Each axis denotes a chemical class, and the radial value represents its normalized relative abundance within each herb. This visualization enables rapid comparison of chemical fingerprints across herbs, highlights dominant compound families and overall chemical diversity, and provides a rationale for prioritizing compound classes for subsequent bioassays and mechanistic studies. Among the 24 compounds, as shown in the radar map (Figure 1C), 15 compounds were from glycyrrhizae radix et rhizome (LR), including 5 flavones, 5 fatty acids, 4 terpenoids, and 1 coumarin. 13 compounds were from chebulae fructus (CF), including 8 phenolic acids, 4 fatty acids, and 1 flavone. 11 compounds were from bovis calculus artifactus (ACB), including 9 steroids and 2 alkaloids. One alkaloid was identified from Indigo Naturalis (NI).

Chebulic acid (peak 2) was taken as a case to introduce identification of compounds. In the negative ion mode, a quasi-molecular ion was detected at m/z 355.0315 [M − H]−, which, respectively, produced the fragment ions at *m/z* 337.0210 [M-H-H_2_O] − and 311.0409 [M-H-CO_2_] − by loss of H_2_O and CO_2_. The compound was confirmed as chebulic acid (25).

### 3.2 Identification of active compounds and illumination of core targets and pathways associated with COVID-19 by network pharmacology analysis

**Figure 2:**
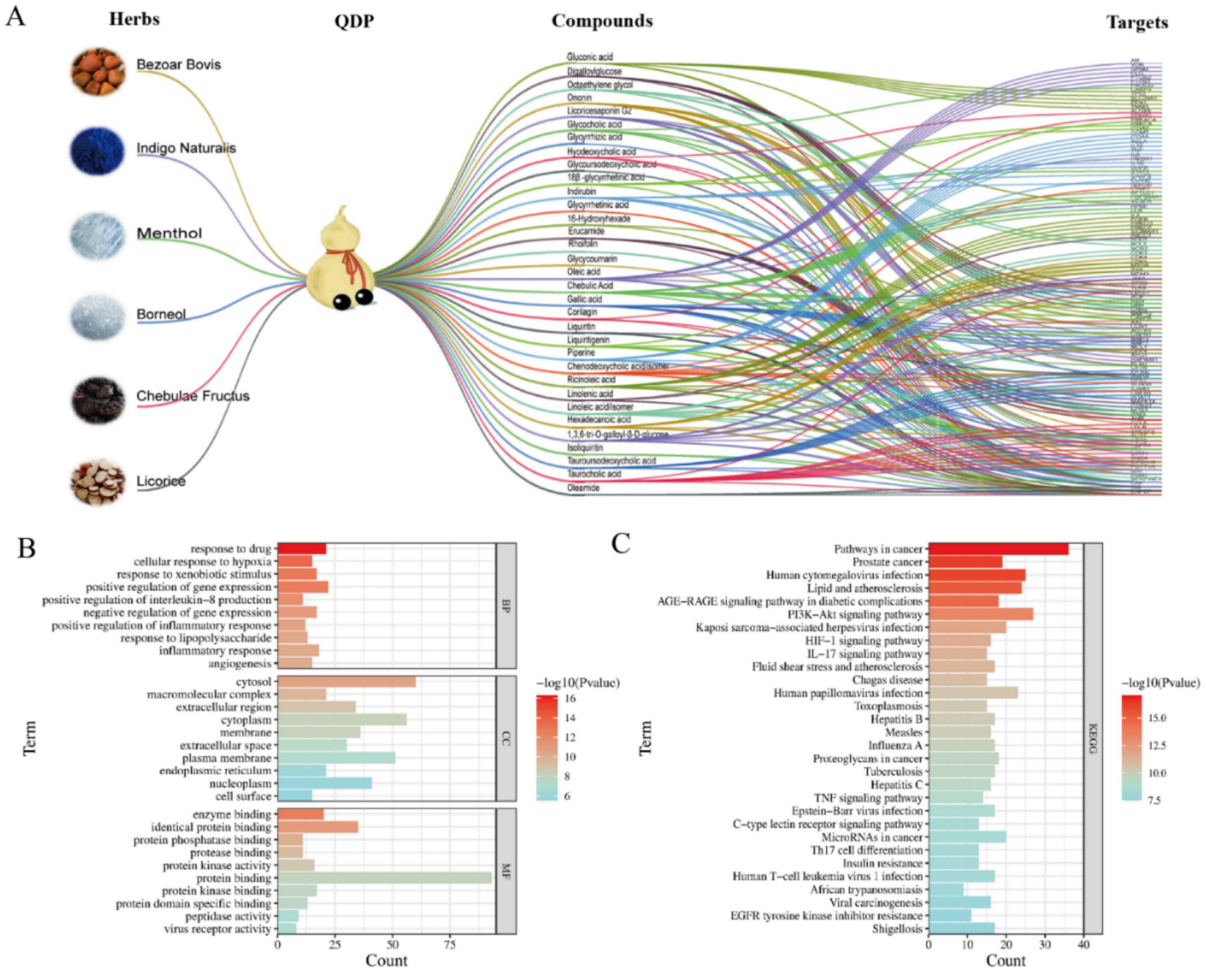
Network pharmacology analysis of herbs-preparation-compounds-targets and functional enrichment. **(A)** Herb-preparation-compound-target network. **(B)** GO enrichment analysis. **(C)** KEGG enrichment analysis.

Focusing on the identified 48 compounds, we employed network pharmacology to preliminarily confirm active compounds in QDP against COVID-19. 468 targets associated with the characterized compounds and 1,958 targets related with COVID-19 were overlapped to obtain the 102 shared targets, which were displayed in the Venn diagram in Supplementary Figures S1A,B. In Figure 2A, the network of herbs-preparation-compounds-targets showed that interactions happened between the complicated compounds and multiple targets to perform antiviral and anti-inflammatory effects, from which 33 compounds were unveiled as the potential active candidates (Supplementary Table S2).

In order to obtain the core targets, the PPI network was constructed according to the shared 102 targets, and the degree value of each target was calculated and ranked to identify core targets. As shown in Supplementary Figure S2C, the higher the degree value was, the more important the node was in the PPI network. The key nodes in the network were considered as the core targets. Accordingly, *β*-actin (ACTB), tumor necrosis factor (TNF), tumor protein p53 (TP 53), vascular endothelial growth factor A (VEGFA), and interleukin-6 (IL-6) were suggested as the core targets against COVID-19.

To further explore the biological functions and pathways, GO and KEGG enrichment analyses were undertaken. As shown in Figure 2B, the top 10 terms were, respectively, ranked by *p*-value and displayed in BP, CC, and MF analyses, from which the important biological functions were enriched, including the response to drug, cellular response to hypoxia, enzyme binding, identical protein binding, and so on. Notably, several immune-related biological processes including IL-8 production, inflammatory response, and response to LPS were significantly enriched (*p* < 0.01). KEGG pathway enrichment analysis indicated that the core targets were enriched in viral infection-related pathways, including human cytomegalovirus infection, Kaposi sarcoma-associated herpesvirus infection, human papillomavirus infection, and so on (Figure 2C). Moreover, as for inflammation regulation, phosphatidylinositol 3-kinase-AKT (PI3K-Akt), interleukin-17 (IL-17), and TNF signaling pathways were suggested as the key pathways.

### 3.3 Exploration in the binding mode of potential active compounds to S^pro^, M^pro^, and PL^pro^ via molecular docking

**Figure 3:**
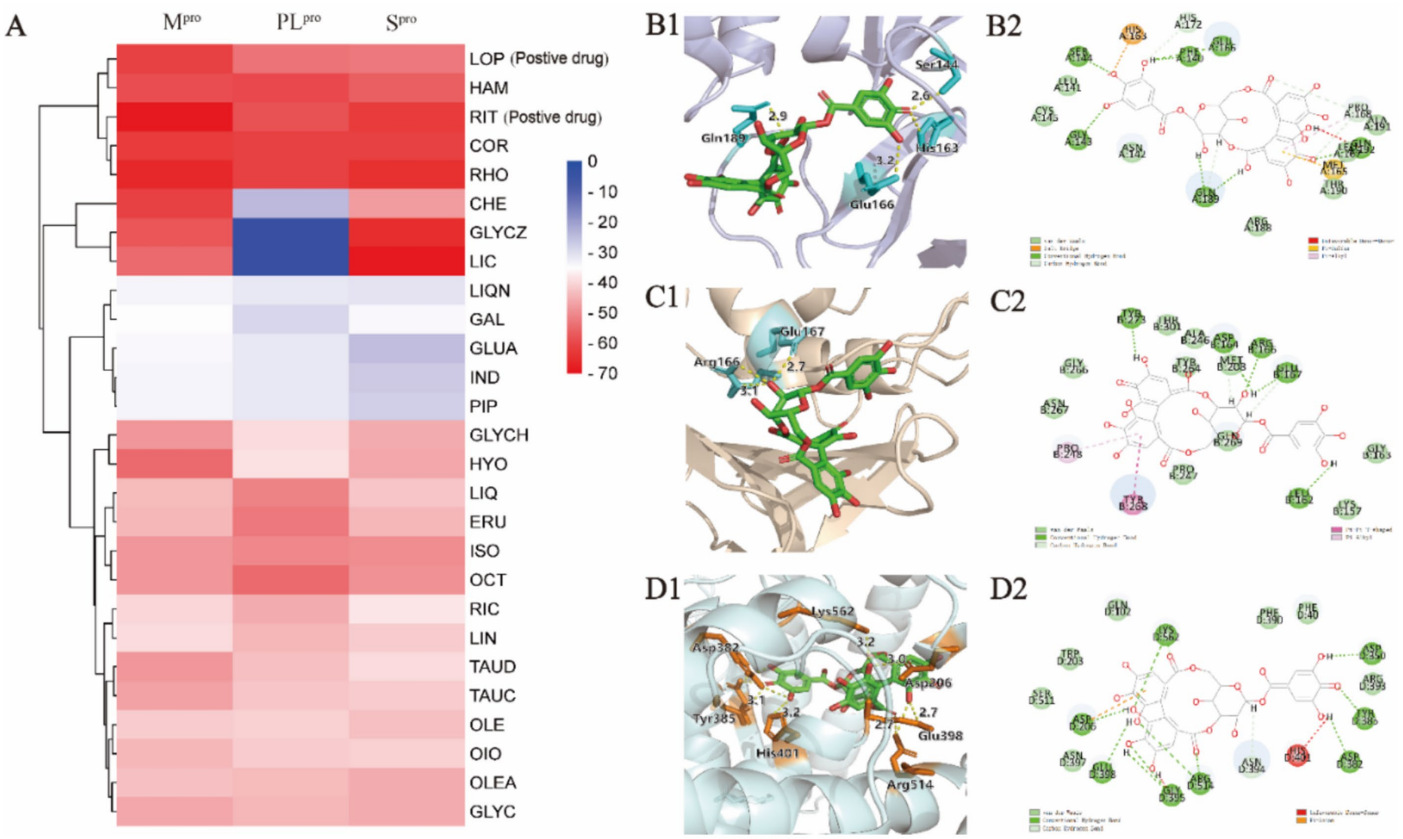
Molecular docking analysis of compounds against viral proteases. **(A)** Heatmap of CDOCKER interaction energies for compounds-M^pro^, compounds-PL^pro^, and compounds-S^pro^ complexes. **(B1,B2)** Binding conformations of COR-M^pro^ complex. **(C1,C2)** Binding conformations of COR-PL^pro^ complex. **(D1,D2)** Binding conformations of COR-S^pro^ complex.

As the key targets for invasion and replication of SARS-CoV-2, S^pro^, M^pro^, and PL^pro^ were employed to dock with the 33 potential active compounds, respectively. Lower cdocker interaction energy indicates the more stable conformation of the target-ligand complex as shown in Table 1. As the original ligand of M^pro^ (PDB:6 LU7), N3 was docked with M^pro^ at a binding energy of −72.88 kcal/mol, while GRL0617, as an original ligand of PL^pro^ (PDB:7CJM), was docked with PL^pro^ at a binding energy of −41.52 kcal/mol (Supplementary Figure S2), resulting in validation of the feasibility of docking model. RIT and LOP, which were reported as the inhibitors of SARS-CoV-2, were proved to have perfect binding with S^pro^, M^pro^, and PL^pro^ as positive drugs. Twenty-six compounds from thirty-three potential bioactive compounds were successfully docked to thees three targets, respectively. As displayed in Figure 3A, the heatmap of binding energy has shown that COR, RHO, and HAM exhibited satisfactory binding with all three targets, whose binding energy was as low as below the score of the original ligand.

**Table 1:** The cdocker interaction energy between the tested compounds and targeted proteins.

No.	Compounds	Abbreviation	The cdocker interaction energy (kcal/mol)		
			M^pro^ (PDB ID: 6 LU7)	PL^pro^ (PDB ID: 7CJM)	S^pro^ (PDB ID: 7T9L)
1	N3 (M^pro^-proligand)	/	−72.88	#	#
2	GRL0617 (PL^pro^-proligand)	/	#	−41.52	#
3	ritonavir (positive drug)	RIT	−70.16	−58.52	−63.15
4	lopinavir (positive drug)	LOP	−61.25	−53.85	−53.13
5	1,3,6-tri-*O*-galloyl-*β*-D-glucose	TGG	−74.39	−64.33	−72.79
6	rhoifolin	RHO	−66.41	−61.72	−65.18
7	corilagin	COR	−62.93	−61.64	−61.72
8	chebulic acid	CHE	−61.21	−23.66	−47.76
9	hamamelitannin	HAM	−59.14	−59.93	−56.25
10	glycyrrhizic acid	GLYCZ	−57.82	#	−65.82
11	licoricesaponin G2	LIC	−55.19	#	−70.19
12	hyodeoxycholic acid	HYO	−55.11	−39.24	−46.76
13	octaethylene glycol	OCT	−49.01	−55.43	−49.72
14	glycocholic acid	GLYCH	−48.90	−39.55	−46.21
15	isoliquiritin	ISO	−48.84	−50.76	−50.37
16	tauroursodeoxycholic acid	TAUD	−48.77	−43.05	−39.64
17	taurocholic acid	TAUC	−47.52	−42.52	−41.65
18	glycycoumarin	GLYC	−46.94	−44.45	−46.00
19	erucamide	ERU	−44.89	−53.11	−44.37
20	ononin	ONO	−44.45	−41.90	−40.80
22	oleic acid	OLEA	−43.04	−44.11	−45.94
23	oleamide	OLE	−41.94	−41.34	−43.15
24	ricinoleic acid	RIC	−40.08	−46.07	−38.60
25	linoleic acid	LIN	−39.43	−44.65	−41.77
26	gallic acid	GAL	−34.95	−28.24	−34.34
27	gluconic acid	GLUA	−33.97	−31.42	−24.26
28	Indirubin	IND	−33.46	−31.24	−26.32
29	liquiritigenin	LIQN	−33.23	−31.51	−30.87
30	piperine	PIP	−33.02	−31.50	−27.25

“#” represents no effective interaction.

COR was taken as an example to display the docking mode with the different targets in Figures 3B1,B2-D1,D2, from which hydrogen bonds and *π*-π stacking were observed. For example, the COR-M^pro^ complex obviously showed the formation of hydrogen bonds, which were formed with the residues of Ser144 (2.6 Å), Glu166 (3.2 Å), and Gln189 (2.9 Å) in the binding site. The interactions between COR and PL^pro^ were witnessed at residues of Glu167 (2.7 Å) and Arg166 (2.8 Å) to form hydrogen bonds. The hydrogen bonds were exhibited in the COR-S^pro^ complex at residues of Asp350 (2.8 Å), Asp382 (3.1 Å), Tyr385 (3.0 Å), Lys562 (3.2 Å), Arg514 (2.7 Å), Glu398 (2.7 Å), and Asp206 (3.0 Å). Moreover, the aromatic ring of COR formed π-π stacking with the side chain residue of Asp206.

For RHO and HAM, we have placed the result graph of the supplementary molecular docking in Supplementary Figure S3. The RHO-M^pro^ complex obviously showed the formation of hydrogen bonds, which were formed with the residues of Glu166 (2.4 Å), Glu166 (2.9 Å), Phe140 (2.0 Å), Phe140 (1.9 Å), Asn142 (2.3 Å), Asn142 (2.8 Å) and Thr26 (1.9 Å) in the binding site. The interactions between RHO and PL^pro^ were witnessed at residues of Thr301 (1.9 Å), Gly163 (2.8 Å) and Asp164 (1.9 Å) to form hydrogen bonds. The hydrogen bonds were exhibited in the RHO-S^pro^ complex at residues of Asp206 (2.0 Å), Asp350 (1.9 Å), Ala348 (2.0 Å) and Ala348 (2.0 Å).

The HAM-M^pro^ complex obviously showed the formation of hydrogen bonds, which were formed with the residues of Thr190 (2.3 Å), Asn142 (2.4 Å) and Cys145 (2.0 Å) in the binding site. The interactions between HAM and PL^pro^ were witnessed at residues of Glu167 (1.9 Å), Tyr268 (3.2 Å), Arg166 (3.3 Å) and Asp164 (2.4 Å) to form hydrogen bonds. The hydrogen bonds were exhibited in the HAM-S^pro^ complex at residues of Asn349 (2.1 Å), Lys562 (1.9 Å) and Lys562 (1.8 Å).

### 3.4 SPR assay for validation of binding affinity with M^pro^, PL^pro^, and S^pro^

**Figure 4:**
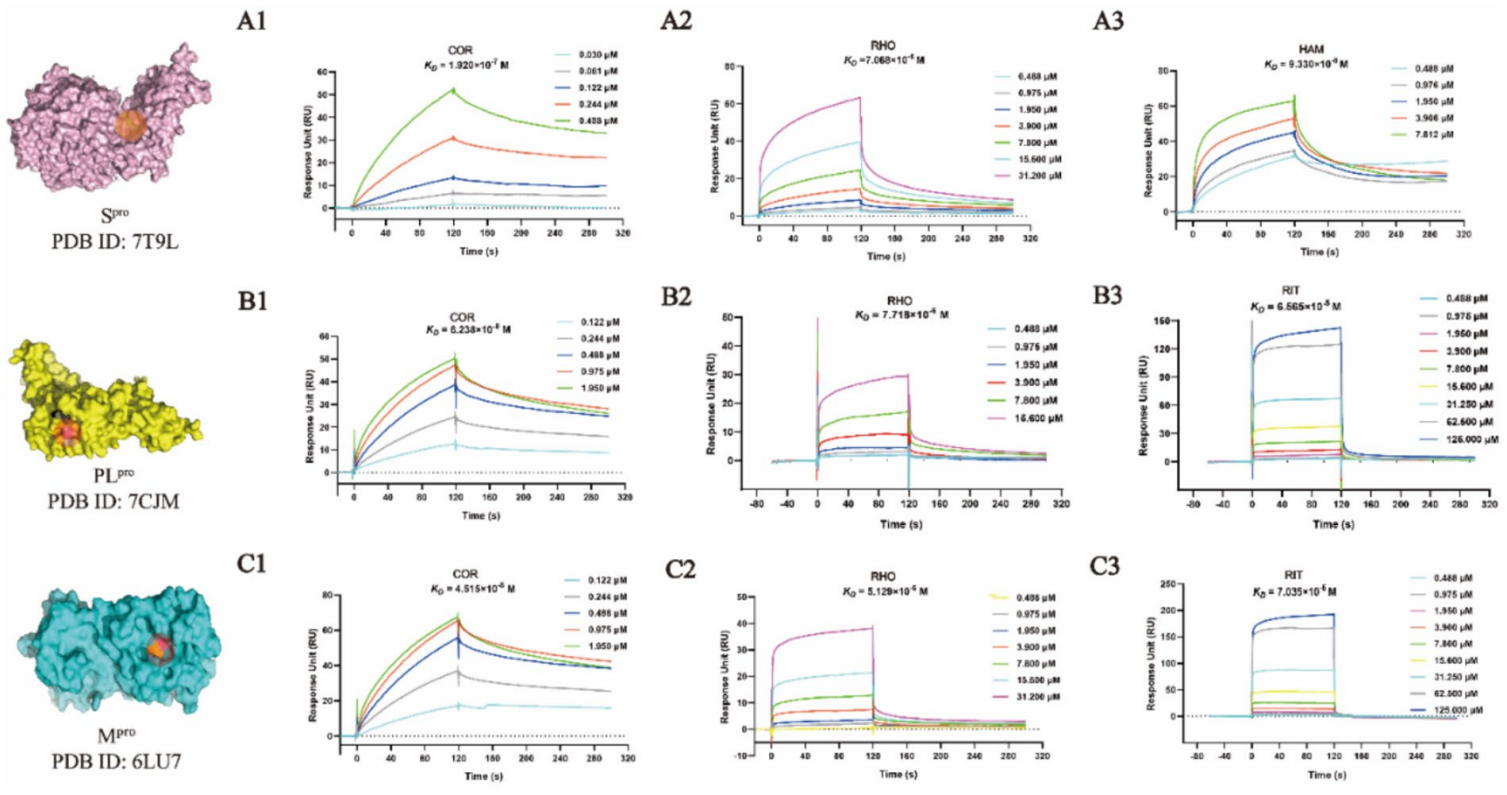
SPR binding affinity analysis of compounds against viral proteases. **(A1-A3)** S^pro^-compound interactions. **(B1-B3)** PL^pro^-compound interactions. **(C1-C3)** M^pro^-compound interactions.

M^pro^ and PL^pro^ were purified with a purify exceeding 95% in our lab as shown in Supplementary Figures S4. By taking the availability of compounds in the market into account, as the representative compounds with the most satisfactory interaction with the focused targets, COR, RHO, and HAM were employed to perform validation of binding affinity by determining the equilibrium dissociation constant (*K_D_*). The lower *K_D_* value of compounds indicates a stronger binding affinity to the targets. In Figures 4A1-A3, targeting S^pro^, the *K_D_* values for COR, RHO, and HAM were determined at 1.920 × 10^−7^ M, 7.068 × 10^−6^ M, and 9.330 × 10^−8^ M, respectively. In comparison, other natural compounds, such as epigallocatechin gallate (*K_D_* = 1.15 × 10^−5^ M), isobavachalcone (*K_D_* = 5.70 × 10^−6^ M) and isochlorogenic acid A (*K_D_* = 1.83 × 10^−5^ M) (26), also demonstrated binding affinity to the S^pro^ RBD and were verified as promising SARS-CoV-2 inhibitors. Therefore, the evaluation of *K_D_* values proves to be an effective strategy for screening potential inhibitors. For PL^pro^ and M^pro^, as illustrated in Figures 4B1-B3,C1-C3, using RIT as a positive drug, the *K_D_* values were evaluated at 6.565 × 10^−5^ M for PL^pro^ and 7.035 × 10^−5^ M for M^pro^. The *K_D_* values for RHO and COR were appraised to fall within the ranges of 4.515 × 10^−8^ M and 7.718 × 10^−6^ M, respectively. Conversely, HAM exhibited negligible affinity toward both PL^pro^ and M^pro^.

Despite the docking results, HAM, RHO and COR showed different binding affinities with M^pro^, PL^pro^, and S^pro^. The virtual screenings, one of the most widely practiced strategies for discovering potential active compounds, presents some design shortcomings. Typically, a scoring function is used to evaluate the strength of the interaction between receptors and ligands. However, this approach often suffers from issues of accuracy and applicability. Furthermore, in virtual screening, the active site is predefined, which may not accurately reflect its true nature. Therefore, while virtual screening could rapidly focus on the potential active compounds and predict their mechanisms, it is imperative to corroborate these predictions with a series of experimental validations.

### 3.5 High-throughput screening inhibitors of M^pro^ and PL^pro^ by FRET

**Figure 5:**
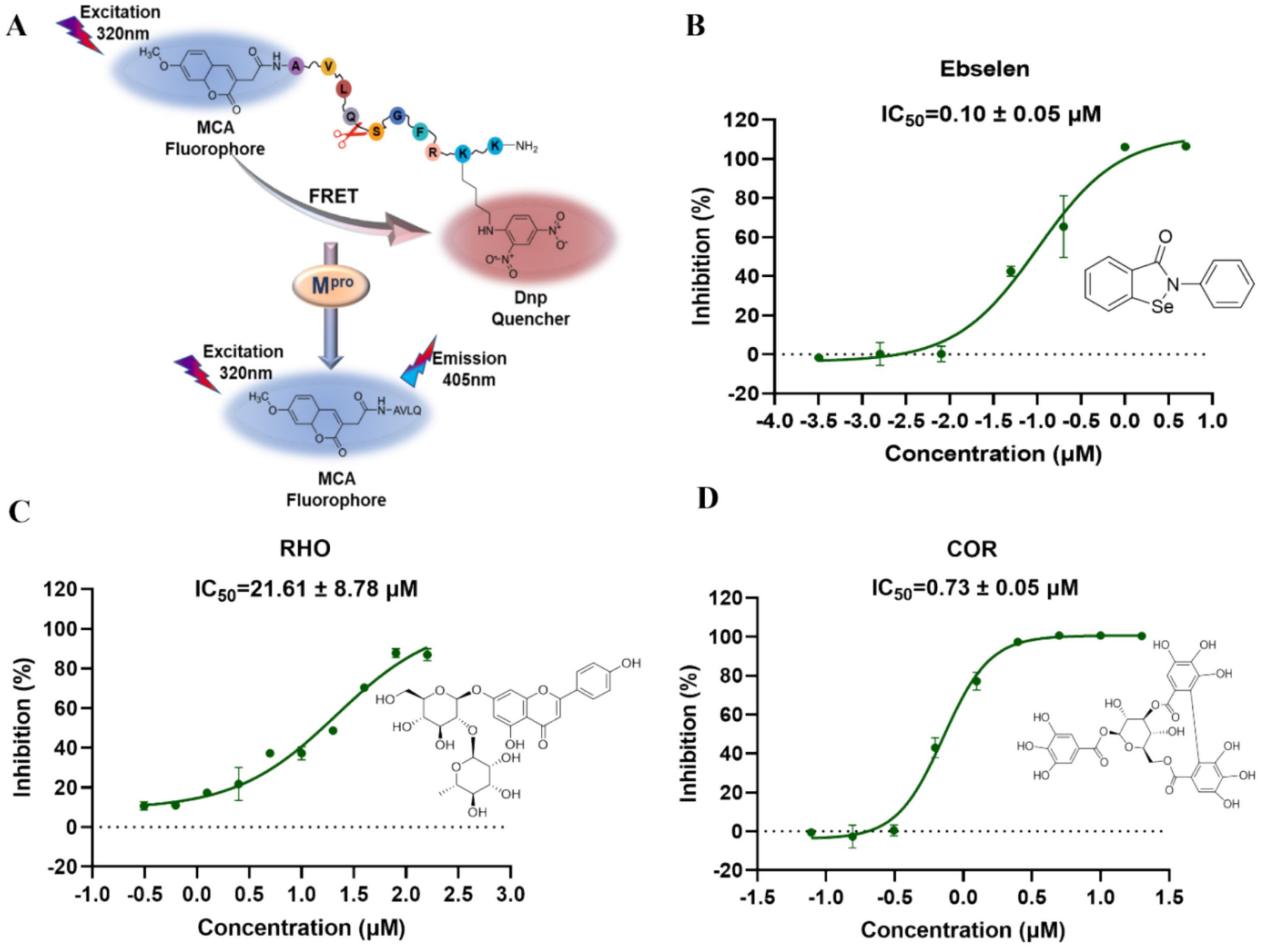
Inhibition activity of the focused compounds on SARS-CoV-2 M^pro^. **(A)** The experimental principle of M^pro^ inhibitory activity. **(B)** The IC_50_ inhibition curve of ebselsen. **(C)** The IC_50_ inhibition curve of RHO. **(D)** The IC_50_ inhibition curve of COR.

FRET has been widely employed in screening the inhibitors of M^pro^ and PL^pro^ because of its high sensitivity and specificity (27). Owing to the excellent binding affinity with M^pro^ and PL^pro^, RHO and COR were further used to evaluate the inhibitory activity on M^pro^ and PL^pro^. As showed in Figure 5, ebselen, as the M^pro^ inhibitor, showed the strongest inhibition on M^pro^ with IC_50_ of 0.10 ± 0.05 μM (Figure 5B). RHO and COR exhibited satisfactory inhibitory activities with IC_50_ of 21.61 ± 8.78 μM (Figure 5C) and 0.73 ± 0.05 μM (Figure 5D). Furthermore, COR demonstrated potent inhibition of the M^pro^ with an IC_50_ value of 0.73 μM, outperforming 1,2,3,4,6-penta-*O*-galloyl-*β*-D-glucose and 1,2,3,6-tetra-*O*-galloyl-*β*-D-glucose with IC_50_ values ranging from 1.33 to 27.37 μM (9). GRL0617, as the PL^pro^ inhibitor, showed inhibitory effects with IC_50_ of 2.69 ± 0.43 μM (Supplementary Figure S5A). Unlike M^pro^, COR and RHO showed poor inhibition on PL^pro^ at 40 μM and IC_50_ beyond 100 μM (Supplementary Figure S5B). The results suggested that COR and RHO exhibited preferentially inhibition activity on M^pro^ by comparing with PL^pro^.

Natural compounds were a rich source to discover the antiviral agents. Due to the safety in clinical practice, the natural products receive more attention. As the natural products, COR and RHO showed significantly inhibitory activity on M^pro^ at a comparable level with the chemical molecules and exhibited specifical inhibitory activity on M^pro^. This difference is related to the structural characteristics of these proteins. In QDP, COR mainly comes from the herb CF, and RHO comes from the herb LR, which were suggested as the main active compounds for antiviral activity by inhibiting M^pro^.

### 3.6 Evaluation of antiviral activity on SARS-CoV-2 omicron BA.5 *in vitro*

**Figure 6:**
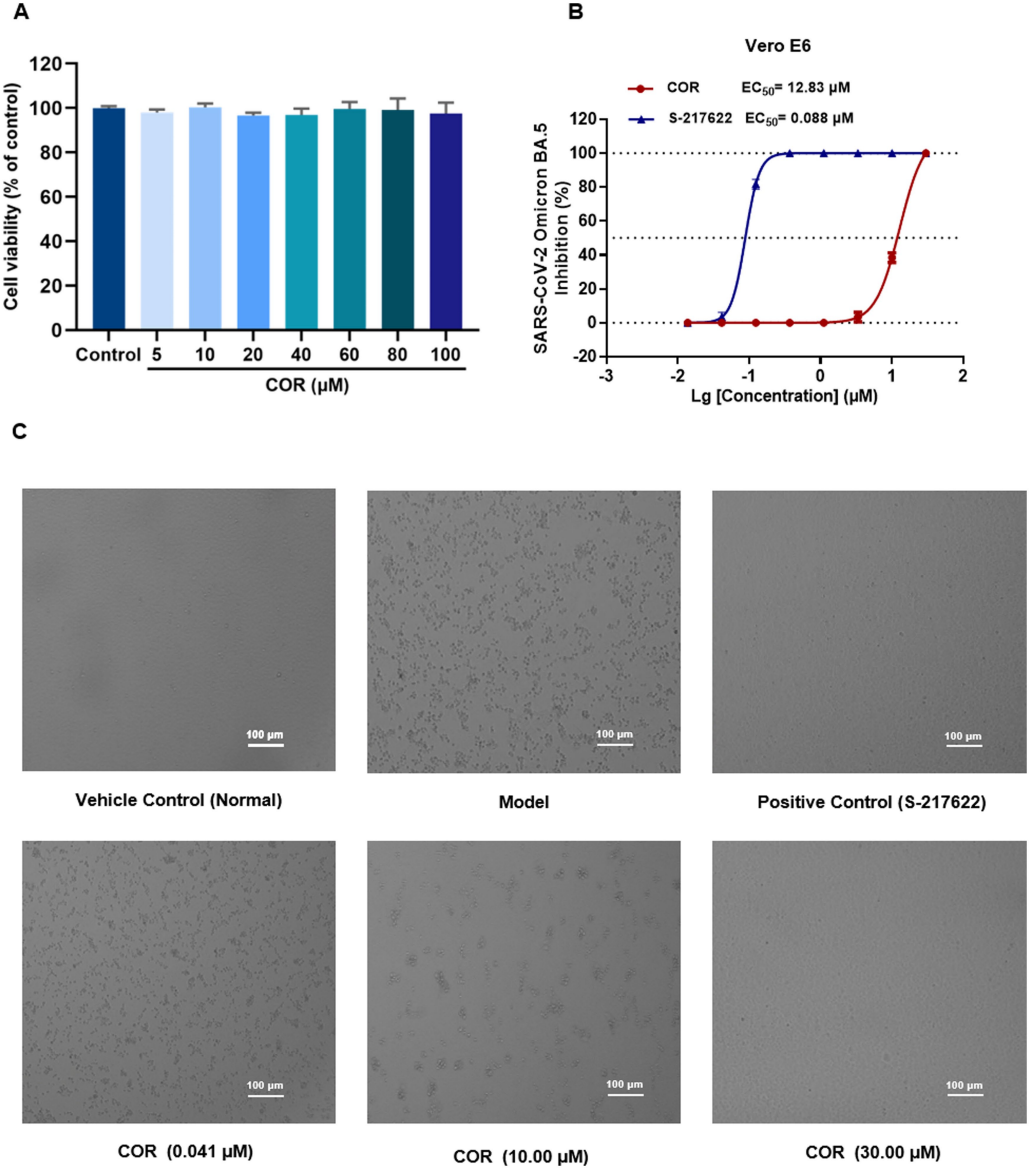
The cytotoxicity and antiviral activity evaluation of COR against SARS-CoV-2 Omicron BA.5. **(A)** Cytotoxicity assessment in Vero E6 cells by MTT assay. **(B)** Inhibition of SARS-CoV-2 Omicron BA.5 replication (mean ± SD, *n* = 3). **(C)** Representative cytopathic effect (CPE) images in Vero E6 cells.

COR exhibited the strongest affinity and inhibitory activity on M^pro^, while *K_D_* of COR with S^pro^ displayed excellent interaction, which was considered as the most potential antiviral compound and employed to perform antiviral validation. In the MTT assay, COR was witnessed to be no cytotoxicity after incubating with Vero E6 cells for 48 h with CC_50_ values more than 100 μM (Figure 6A).

S-217622, the first oral non-covalent and non-peptidic SARS-CoV-2 M^pro^ inhibitor, is used as a positive control from the clinical candidate (28). S-217622 and COR was diluted into the different concentrations and incubated with SARS-CoV-2 Omicron BA.5 variant in Vero E6 cells, respectively. As shown in Figure 6B, a dose-dependent manner of COR was observed by protecting the cells damaged by SARS-CoV-2 Omicron BA.5. The EC_50_ of COR was obtained at 12.83 μM and that of S-217622 was 0.088 μM, showing the excellent antiviral activity. Representative images of CPE formation were displayed in Figure 6C. We speculated that COR possibly exerts antiviral activity through S^pro^, M^pro^, and PL^pro^ to interfere with the viral invasion and replication (29).

As a phenolic acid, COR derives from CF in QDP, which has been proved to inhibit the activity of reverse transcriptase of RNA tumor viruses and show negligible toxicity on normal cells and tissues (30). In this study, COR was screened from the identified compounds library in QDP by an integrated strategy of dry and wet method, which displays specifically inhibitory activity on M^pro^. Additionally, COR has a good solubility and stability, which is considered as a promising scaffold for developing inhibitors against coronavirus (31).

### 3.7 Evaluation of anti-inflammatory activity *in vitro*

**Figure 7:**
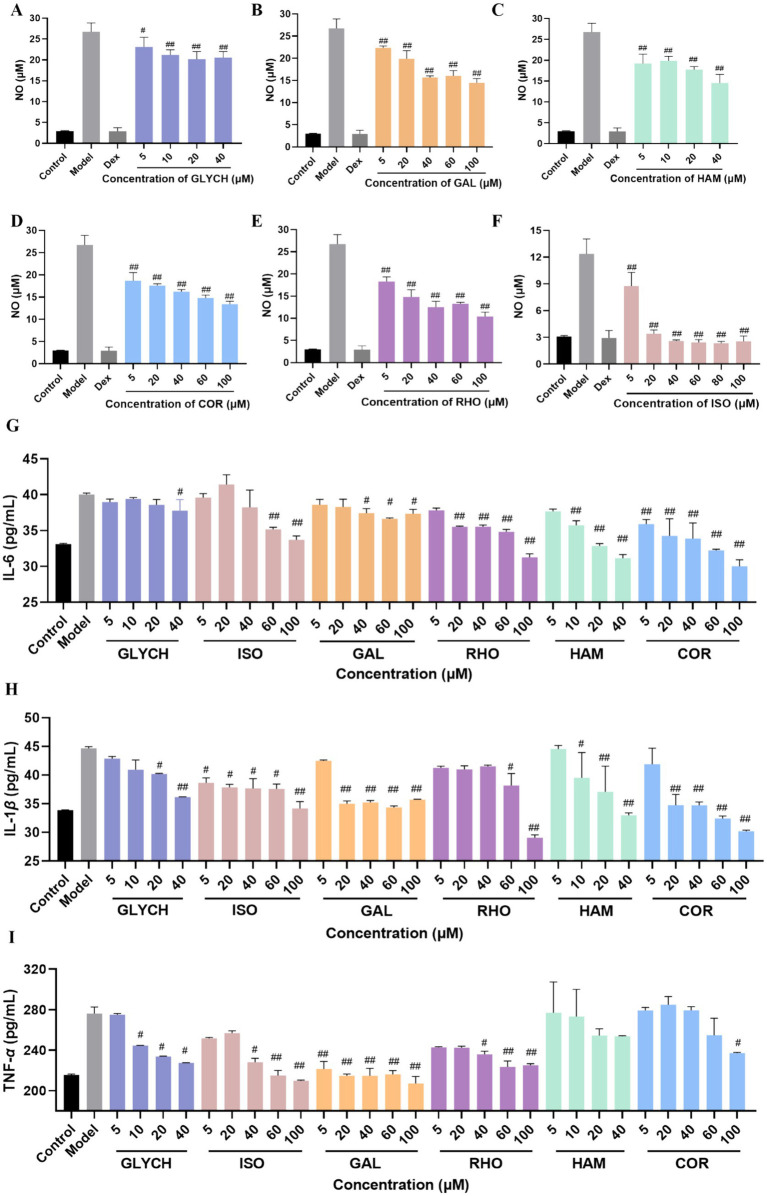
Anti-inflammatory activity evaluation of the tested compounds by the level of NO **(A–F)**, IL-6 (G), IL-1 *β* **(H)**, and TNF-*α* **(I)**. (mean ± SD, *n* = 3). *p*^#^ < 0.05 and *p*^##^ < 0.01 vs. model group.

As the main lethal factors of COVID-19, the fatal pneumonia was caused by the cytokine storm. Anti-inflammatory therapy is considered as an effective strategy for reducing the damage on the important organs (32). In order to screen the active compounds in QDP with anti-inflammatory activity, 24 compounds from 33 potential active compounds, which accessibly were deserved from market, were used to verify the anti-inflammatory effects *in vitro* by evaluating the NO, IL-6, IL-1*β*, and TNF-*α* level in LPS-induced RAW 264.7 cells. To establish the LPS stimulation condition, a preliminary dose–response experiment was conducted in RAW 264.7 cells. Cells were treated with LPS at 1, 2, 5, or 10 μg/mL, and NO levels in the culture supernatants were measured by the Griess method using a kit-derived standard curve (Supplementary Figure S6A). All LPS concentrations significantly increased NO compared with the blank control, with no significant differences among the LPS groups (Supplementary Figure S6B), indicating a response plateau from 1 to 10 μg/mL. Therefore, 1 μg/mL-the lowest dose achieving a significant response-was chosen for subsequent experiments to ensure consistent activation while minimizing potential nonspecific effects. As shown in Supplementary Figure S7, RHO, COR, GAL, and ISO exhibited no cytotoxicity up to 100 μΜ in RAW264.7 cells (CC_50_ > 100 μΜ), while for HAM and CLYCH, cell viability significantly decreased (*p* < 0.05) above 40 μΜ. When the RAW264.7 cells were exposed to LPS (1 μg/mL), NO level increased significantly. As shown in Figures 7A–F, comparing model group, the production of NO can be inhibited by RHO, COR, HAM, GAL, ISO, and GLYCH within the tested concentration in the dose-dependent manners.

Furthermore, the main inflammatory factors were tested at different concentrations. As displayed in Figures 7G–I, RHO, COR, HAM, GAL, ISO, and GLYCH can significantly reduce the level of IL-6 and IL-1*β*. Except for HAM, RHO, COR, GAL, ISO, and GLYCH can also decrease the level of TNF-*α*.

Generally, through this study, by integrating of *in silico* screening and bioactivity validation, the features of QDP were unveiled to combat COVID-19 via the multi-compounds, multi-targets, and multi-pathways. Also, the focused compounds can interfere with viral invasion, viral replication, and cytokine storm, showing satisfactory performance in multi-stage of COVID-19.

## 4 Discussion

QDP is officially recommended in the “Home Care Guideline for COVID-19 Patients” issued by the Joint Prevention and Control Mechanism of the State Council of China. Its real-world clinical evidence supports the effectiveness of QDP in pharyngitis and related inflammatory conditions. Clinical studies report significant improvements in sore throat, pharyngeal dryness, and swelling in patients with acute or chronic pharyngitis, with a rapid onset of action and favorable patient adherence (33, 34). Moreover, pharmacokinetic studies in rats show that key compounds such as borneol and menthol are rapidly absorbed after oral administration (T_max_: 20–25 min), which is consistent with the prompt symptom relief observed in clinical settings and may support QDP’s therapeutic effects (8).

The COVID-19 pandemic has exerted unparalleled impacts on global health and economic stability. The onging emergence of SARS-CoV-2 variants continues to pose a significant public health risk, manifesting with severe respiratory symptoms, serious complications like viral neumonia, long-term ramifications known as “Long COVID” (32) and elevated mortality rates especially among vulnerable populations. Notably, recent genomic surveillance data (GISAID, 2024) highlight that variants like JN.1 carry mutations in PL^pro^ such as R168H, which experimentally confer resistance to clinical protease inhibitors by distorting the conformation of S3/S4 subpocket (35). This underscores the urgent need for broad-spectrum therapeutics targeting conserved viral elements.

As crucial targets in the viral lifecycle, S^pro^, M^pro^, and PL^pro^ play key roles in viral entry and replication. S^pro^ facilitates the identification of ACE2 receptors, while PL^pro^ and M^pro^ are involved in cleaving of polyproteins. Although both M^pro^ and PL^pro^ are enssential for viral replication within the host, their catalytic Mechanisms are distinct. M^pro^ operates through a catalytic dyad (Cys145-His41), cleaving polyproteins at glutamine (P1) residues within a conserved substrated-binding groove comprising four subpockets (S1’, S1, S2, S3/S4). Structural studies confirm that the S2 subpocket displays pronounced hydrophobicity, accommodating bulky P2 residues such as leucine (36). In contrast, PL^pro^ relies on a catalytic triad (Cys111-His272-Asp286) and a zinc-binding domain essential for structural integrity. PL^pro^ exhibits dual deubiquitinating and deISGylating activities through extended substrate-recognition grooves, with allosteric modulation via C270 critically regulating catalytic efficiency (37).

These mechanistic distinctions necessitate tailored therapeutic strategies, particularly given PL^pro^’s higher mutational flexibility compared to the evolutionarily constrained M^pro^ active site (38, 39). Critically, PL^pro^ cleaves both ubiquitin (Ub) and interferon-stimulated gene 15 (ISG15) from host proteins, disrupting innate immune signaling pathways. This immune evasion occurs via the direct cleavage of STING, which abolishes TBK1 phosphorylation and IRF3 nuclear translocation—a mechanism absent in M^pro^, which exclusively processes viral polyproteins (40). This functional distinction categorizes PL^pro^ as a dual-function viral protease and immune modulator.

Cryo-EM structures demonstrate that PL^pro^’s BL2 loop undergoes hinge-like movements to accommodate bulky Ub/ISG15 substrates, while its zinc finger domain maintains structural integrity via Zn^2+^ chelation (40). Distinct from M^pro’^s compact active site, PL^pro^ features extended substrate-binding grooves (including BL2 loop and Ub-binding region) and dual enzymatic activities create pharmacological barriers insurmountable by M^pro^-targeted compounds. Moreover, divergent electrostatic potentials govern substrate recognition: M^pro^’s S1 pocket is characterized by electronegativity (mediated by His163/Glu166), whereas PL^pro^’s Ub-binding region features cationic residues (Lys157/Arg166) (41). Therefore, developing inhibitors for PL^pro^ is more challenging.

In the quest for effective COVID-19 treatments, TCM has demonstrated considerable efficacy in managing symptoms and enhancing recovery, leveraging its diverse array of bioactive compounds. These compounds are noted for their direct antiviral, anti-inflammatory, and immunomodulatory properties. The structural diversity of phytochemicals present in Chinese herbs highlights their promise for the development of innovative antiviral therapies, which are capable of both neutralizing the virus and modulating excessive inflammatory reactions (42). Importantly, the TCM formulation known as QDP, traditionally employed in treating pharyngitis, has been endorsed by the Joint Prevention and Control Mechanism of the State Council as an effective remedy for at-home treatment of COVID-19, as outlined in the guidelines published in December 2022. This recommendation aligns with the WHO’s 2023 guidelines on integrating traditional medicines into pandemic preparedness, though it calls for standardized quality control of herbal preparations.

Currently available anti-coronavirus TCMs, such as Lianhua Qingwen capsules (43) and Huashi Baidu formula (44), rely heavily on the antiviral and anti-inflammatory effects of compounds. The volatile compounds borneol and isoborneol in QDP provide immediate mucosal permeability. The non-volatile compounds, berberine, alkaloids and so on, sustained the anti-inflammatory and antiviral activities (45, 46). The integration of volatile and non-volatile compounds forms a unique fast-acting and long-lasting synergy, highlighting the chemical uniqueness of QDP. This chemical composition may potentially translate into multiple pharmacological advantages. The unique troche formulation and volatile compounds enable direct action at the throat and mouth, possibly offering faster local immune regulation and physical barrier reinforcement. Studies have shown that both chebulagic acid and punicalagin, at noncytotoxic concentrations, can reduce virus-induced plaque formation in Vero E6 monolayers. These compounds appear to function as allosteric regulators, targeting SARS-CoV-2 M^pro^ (43). Corilagin binds directly to RdRp, robustly inhibiting its polymerase activity, as evidenced by both cell-free and cell-based assays (47). Tannic acid may contribute to the treatment of inflammation by decreasing MPO enzyme activity (48).

Our investigation employed a dual strategy, integrating *in silico* screening and bioactivity assays, to discover and validate the therapeutic potential of QDP against COVID-19. Notably, compared with the single-step screening methods, this “dry-wet combined” strategy not only saves the use of standards and solvents, but also significantly accelerates the identification speed of bioactive candidates. In the face of emerging and sudden diseases, it can quickly screen out potential effective components. This research represents the first isolation and characterization of chemical compounds in QDP. Among the 48 identified compounds, network pharmacology analysis revealed likely active compounds and elucidated their mechanisms of action against COVID-19. We targeted key proteins, including S^pro^, M^pro^, and PL^pro^, performing exhaustive virtual screenings of QDP to assess its inhibitory capabilities against SARS-CoV-2. Subsequently, we verified the inhibitory interactions of three principal phytocompounds—COR, RHO, and HAM—through molecular docking studies. Moreover, these compounds demonstrated consistent inhibitory effects on M^pro^ and exhibited anti-inflammatory properties.

Among the compounds examined, COR, a polyphenolic gallotannin recognized for its anti-inflammatory and antioxidant properties, emerged as a notably potent agent (49). It displayed a strong binding affinity to S^pro^, M^pro^, and PL^pro^, evidenced by a low *K_D_*. Functional assays further confirmed its efficacy, with COR significantly inhibiting M^pro^ in FRET assay (IC_50_ = 0.73 ± 0.05 μM) and demonstrating antiviral efficacy in Vero E6 cells infected with the SARS-CoV-2 Omicron BA.5 variant (EC_50_ = 12.83 μM). Additionally, COR reduced inflammatory cytokine production, indicating a dual role in both viral inhibition and immune modulation. Given its broad-spectrum activity against other pathogens, including influenza (50) and herpes simplex viruses, COR represents a promising candidate for further antiviral therapeutic exploration.

RHO, another potential agent, is a flavonoid glycoside known for its anti-inflammatory, antioxidant, and anticancer properties. However, it exhibited a limited activity in our assays (51). While RHO was effective against M^pro^, it failed to inhibit the SARS-CoV-2 Omicron BA.5 variant. This specificity highlights the necessity for deeper molecular investigations to fully ascertain RHO’s mechanistic roles and its potential therapeutic utility against SARS-CoV-2.

From a therapeutic standpoint, these findings are encouraging, offering a robust scientific foundation for the clinical application of QDP in treating COVID-19. A thorough examination of the interactions and the required inhibitory concentrations can inform dosage and formulation strategies in clinical contexts. Nonetheless, translating these results into clinical practice necessitates comprehensive clinical trials to ascertain efficacy and safety thoroughly.

In addition to the virus invasion inhibition investigated in this work, we plan to further explore the effects of QDP on other key stages of the viral life cycle, including replication, assembly, and release. Moreover, more complex models such as organoids will be employed to systematically evaluate the antiviral immune regulatory effects of the drug in a simulated human respiratory microenvironment.

Overall, this study not only underscores the therapeutic potential of TCM addressing COVID-19 but also contributes to the global quest for antiviral drug discovery by identifying novel bioactive compounds. Future research should focus on validating these findings in clinical settings and elucidating the mechanistic pathways of these compounds to maximize their therapeutic potential.

## 5 Conclusion

As endorsed by the National Health Commission of the People’s Republic of China for COVID-19 treatment, QDP was comprehensively investigated through multiple scientific approaches. The study involved the clarification of chemical compounds, network pharmacology analysis, molecular docking simulations, binding affinity assessments by SPR, enzymatic activity measurements via FRET, and evaluations of antiviral and anti-inflammatory activities. This integrated virtual and experimental strategy enabled the rapid screening of components, identifying COR as exceptionally potent in antiviral capacity, and RHO, COR, HAM, GAL, ISO, and GLYCH as significant anti-inflammatory agents. In general, this study provides a strategy and procedures for rapid and effective identification of active compounds and elucidation of their mechanism, conducing to the scientific application of Chinese patent medicine in treating COVID-19 patients. Furthermore, this study underscores the critical role of integrating traditional medicine with contemporary scientific techniques to explore potential therapies for emerging infectious diseases.

## Data Availability

The datasets presented in this study can be found in online repositories. The names of the repository/repositories and accession number(s) can be found in the article/Supplementary material.

## Footnotes

^1^ https://tcmspw.com/tcmsp.php

^2^ http://www.swisstargetprediction.ch/

^3^ https://www.genecards.org/

^4^ https://www.omim.org/

^5^ https://david.ncifcrf.gov/

^6^ https://pubchem.ncbi.nlm.nih.gov/

^7^ https://www.rcsb.org/
