# Supplementary material for: Elucidation of anti-SARS-CoV-2 and anti-inflammatory bioactives in Qingyan Dropping Pills via integrated in silico screening and bioactivity validation
Source: Front Med (Lausanne). 2025 Nov 10;12:1684713. doi: 10.3389/fmed.2025.1684713 (PMC12640959; doi:10.3389/fmed.2025.1684713)
Supplement: Supplementary file 1 [file Presentation_1.pdf]

Supplementary information

Elucidation of anti-SARS-CoV-2 and anti-inflammatory bioactives in Qingyan Dropping Pills via integrated *in silico* screening and bioactivity validation

Liting Liu [1,†], Xinru Li [1,†], Xinyue Wang [1], Peng Zhang [1], Qi Yang [2], Tong Geng [3], Yuefei Wang [1,4], Junhua Zhang [1,4], Changjian Wang [1*], Jing Yang [1*], Min Zhang [1,4*]

[1]State Key Laboratory of Chinese Medicine Modernization, Tianjin University of Traditional Chinese Medicine, 301617, Tianjin, China

[2]Guangzhou Laboratory, 510005, Guangzhou, China

[3]Tianjin Pharmaceutical Da Ren Tang Group Corp., Ltd. Traditional Chinese Medicine Research Institute, 300171, Tianjin, China

[4]Haihe Laboratory of Modern Chinese Medicine, 301617, Tianjin, China

***Correspondence**:
CJ Wang; J Yang; M Zhang
wangchangjian23@163.com (C Wang); yangjingoffice@163.com (J Yang);
zhangm036@tjutcm.edu.cn (M Zhang)

[†] These authors contributed equally to this work.

Keywords: COVID-19; SARS-CoV-2; Qingyan Dropping Pills (QDP); antivirus; anti-inflammation.

Table S1 The identified compounds in QDP by UPLC-Q/TOF-MS

NO.	t_R (min)	The negative/positive ion mode (m/z) MS	MS/MS	Error (ppm)	Formula（Molecular weight）	Identification	References
1	1.533	195.0531$[M-H]^-$	–	−10.63	$C_6H_{12}O_7$ (196.155)	gluconic acid	–
2*	2.002	355.0315$[M-H]^-$	337.0210$[M-H-H_2O]^-$ 311.0409$[M-H-CO_2]^-$	−2.3	$C_{14}H_{12}O_{11}$ (356.238)	chebulic acid	[1]
3*	2.072	169.0130$[M-H]^-$	125.0235$[M-H-CO_2]^-$	7.38	$C_7H_6O_5$ (170.120)	gallic acid	[2]
4	3.005	127.0392$[M+H]^+$	109.0289$[M+H-H_2O]^+$	−1.81	$C_6H_6O_3$ (126.110)	maltol	–
5	3.006	483.0784$[M-H]^-$	331.0688[M-H-galloyl]$^-$	−0.77	$C_{20}H_{20}O_{14}$ (484.364)	digalloylglucose /isomer	[1]
6	3.274	483.0791$[M-H]^-$	313.0573[M-H-galloyl-$H_2O]^-$	−2.22	$C_{20}H_{20}O_{14}$ (484.364)	digalloylglucose /isomer	[1]

			169.0132[M-H-galloyl-Glcr]$^-$				
7*	3.609	483.0795[M-H]$^-$	331.0670[M-H-galloyl]$^-$ 169.0136[M-H-galloyl-Glcr]$^-$	−3.05	$C_{20}H_{20}O_{14}$ (484.364)	digalloylglucose /isomer	[1]
8	4.078	383.0618[M-H]$^-$	337.0204[M-H-HCOOH]$^-$	0.48	$C_{16}H_{16}O_{11}$ (384.292)	2-*O*-feruloylhydroxycitric acid/ isomer	–
9	4.346	785.0838[M-H]$^-$	633.0731[M-H-galloyl]$^-$	2.92	$C_{34}H_{26}O_{22}$ (786.557)	tellimagrandin I	[1]
10	4.614	383.0618[M-H]$^-$	337.0175[M-H-HCOOH]$^-$	7.01	$C_{16}H_{16}O_{11}$ (384.292)	2-*O*-feruloylhydroxycitric acid/ isomer	–
11	4.949	417.1180[M-H]$^-$	399.0581[M-H-H_2O]$^-$	4.09	$C_{21}H_{22}O_9$ (418.394)	liquiritin isomer	[3]
12*	5.484	633.0748 [M-H]$^-$	300.9992[M-H-galloylglucose]$^-$ 169.0139[M-H-Glcr-HHDP]$^-$	−2.31	$C_{27}H_{22}O_{18}$ (634.453)	corilagin	[2]

13	5.617	371.2282[M+H]$^+$ 393.2105[M+Na]$^+$	–	−2.27	$C_{16}H_{34}O_9$ (370.436)	octaethylene glycol	–
14	6.020	635.0892[M-H]$^-$	483.0825[M-H-galloyl]$^-$	−1.28	$C_{27}H_{24}O_{18}$ (636.469)	1,3,6-tri-*O*-galloyl-*β*-D-glucose	[1]
15	6.288	787.1019[M-H]$^-$	635.0902[M-H-galloyl]$^-$	4.00	$C_{34}H_{28}O_{22}$ (788.573)	1,2,3,4-tetragalloylglucose	[1]
16*	7.092	577.1558[M-H]$^-$	–	0.83	$C_{27}H_{30}O_{14}$ (578.518)	rhoifolin	–
17*	7.427	417.1098[M-H]$^-$	255.0576[M-H-Glcr]$^-$ 135.0017[M-H-C_8H_8O]$^-$	22.31	$C_{21}H_{22}O_9$ (418.394)	liquiritin	–
18*	8.096	417.1124[M-H]$^-$	255.0563[M-H-Glcr]$^-$ 135.0028[M-H-C_8H_8O]$^-$	16.08	$C_{21}H_{22}O_9$ (418.394)	isoliquiritin	–
19*	8.230	475.1276[M-H+HCOOH]$^-$	267.0666[M-H-Glcr]$^-$	−13.92	$C_{22}H_{22}O_9$ (430.405)	ononin	[4]
20*	8.565	255.0613[M-H]$^-$	135.0024[M-H-C_8H_8O]$^-$ 119.0437[M-H-$C_7H_4O_3$]$^-$	6.99	$C_{15}H_{12}O_4$ (256.253)	liquiritigenin	[5]

21	8.833	514.2877[M-H]$^-$<br>1029.5776[2M-H]$^-$	–	−6.42	$C_{26}H_{45}NO_7S$ (515.703)	taurocholic acid	[6]
22*	9.101	837.3906[M-H]$^-$	819.3829[M-H-H_2O]$^-$	0.98	$C_{42}H_{62}O_{17}$ (838.932)	licoricesaponin G2	[7]
23*	9.235	464.3022[M-H]$^-$<br>929.6107[2M-H]$^-$	–	−0.94	$C_{26}H_{43}NO_6$ (465.623)	glycocholic acid	[6]
24*	9.235	821.3984[M-H]$^-$	645.3650[M-H-GlcAr]$^-$<br>469.3345[M-H-2GlcAr]$^-$	−2.3	$C_{42}H_{62}O_{16}$ (822.932)	glycyrrhizic acid	[8]
25*	9.771	498.2921[M-H]$^-$<br>500.3134[M+H]$^+$	–	1.17	$C_{26}H_{45}NO_6S$ (499.70)	tauroursodeoxycholic acid	[9]
26*	9.971	407.2783[M-H]$^-$	371.1471[M-H-2H_2O]$^-$	4.91	$C_{24}H_{40}O_5$ (408.57)	ansocholic acid	[9]
27*	10.038	407.2805[M-H]$^-$<br>815.5754[2M-H]$^-$	–	−2.59	$C_{24}H_{40}O_5$ (408.57)	cholic acid	[6]

28*	10.172	391.2860[M-H]$^-$ 437.2912[M-H+HCOOH]$^-$ 783.5824[2M-H]$^-$	–	−1.58	$C_{24}H_{40}O_4$ (392.572)	hyodeoxycholic acid	[6]
29*	10.373	450.3252[M+H]$^+$ 899.6378[2M+H]$^+$	–	−8.44	$C_{26}H_{43}NO_5$ (449.623)	glycoursodeoxycholic acid	[6]
30	10.373	367.1204[M-H]$^-$	–	0.3	$C_{21}H_{20}O_6$ (369.380)	glycycoumarin	[10]
31	10.574	645.3673[M-H]$^-$	483.3104[M-H-Glcr]$^-$	−1.98	$C_{36}H_{54}O_{10}$ (646.81)	18*β*-glycyrrhetinic acid-3-*O*-*β*-D-glucoside	–
32*	10.640	286.1450[M+H]$^+$	–	−4.3	$C_{17}H_{19}NO_3$ (285.338)	piperine	–
33*	10.909	261.0670[M-H]$^-$ 263.0823[M+H]$^+$	–	0	$C_{16}H_{10}N_2O_2$ (262.263)	indirubin	[11]
34	11.378	391.2860[M-H]$^-$ 437.2917[M-	–	−1.58	$C_{24}H_{40}O_4$ (392.572)	chenodeoxycholic acid/isomer	[9]

		H+HCOOH]$^-$<br>783.5792[2M-H]$^-$					
35*	11.597	391.2871[M-H]$^-$<br>783.5767[2M-H]$^-$	–	−4.39	$C_{24}H_{40}O_4$ (392.572)	chenodeoxycholic acid/isomer	[9]
36	12.649	342.2051[M+H]$^+$	–	3.71	$C_{21}H_{27}NO_3$ (341.444)	(2*E*,8*E*)-9-(1,3-benzodioxol-5-yl)-1-piperidin-1-ylnona-2,8-dien-1-one	–
37	12.917	496.3413[M+H]$^+$	–	−3.09	$C_{24}H_{50}NO_7P$ (495.630)	1-palmitoyl-lysophosphatidylcholine/isomer	–
38	13.186	297.2441[M-H]$^-$	269.2112[M-H-CO]$^-$	−1.96	$C_{18}H_{34}O_3$ (298.461)	ricinoleic acid	–
39*	13.454	469.3353[M-H]$^-$<br>471.3482[M+H]$^+$	–	−6.32	$C_{30}H_{46}O_4$ (470.684)	glycyrrhetinic acid	[12]
40	16.200	277.2182[M-H]$^-$	248.9723[M-H-CH_2CH_3]$^-$	−3.23	$C_{18}H_{30}O_2$ (278.430)	linolenic acid	–

41*	16.669	271.2289[M-H]$^-$	243.8893[M-H-CO]$^-$	−3.8	$C_{16}H_{32}O_3$ (272.432)	16-hydroxyhexadecanoic acid	–
42	17.206	496.3426[M+H]$^+$	478.3316[M+H-H_2O]$^+$	−3.09	$C_{24}H_{50}NO_7P$ (495.630)	1-palmitoyl-lysophosphatidylcholine/isomer	–
43*	17.471	282.2803[M+H]$^+$	–	−4.11	$C_{18}H_{35}NO$ (281.477)	oleamide	–
44	17.941	279.2348[M-H]$^-$	–	−6.61	$C_{18}H_{32}O_2$ (280.445)	linoleic acid/isomer	–
45	18.209	279.2341[M-H]$^-$	–	−4.1	$C_{18}H_{32}O_2$ (280.445)	linoleic acid/isomer	–
46	19.615	255.2342[M-H]$^-$	–	−4.88	$C_{16}H_{32}O_2$ (256.424)	hexadecanoic acid	[13]
47	19.883	281.2498[M-H]$^-$	–	−5.32	$C_{18}H_{34}O_2$ (282.461)	oleic acid	[13]
48*	23.901	338.3427[M+H]$^+$ 675.6742[2M+H]$^+$	–	0	$C_{22}H_{43}NO$ (337.583)	erucamide	–

* Compared with standards. Glcr, Glucose residue; HHDP, Hexahydroxydibenzoyl; GlcAr, Glucuronic Acid residue.

Table S2 The degree value of potential active compounds obtained by analysis of network pharmacology

No.	Compounds	Abbreviation	Degree
1	ricinoleic acid	RIC	14
2	1,3,6-tri-*O*-galloyl-*β*-D-glucose	TGG	14
3	rhoifolin	RHO	13
4	18*β*-glycyrrhetinic acid-3-*O*-*β*-D-glucoside	GLYAG	13
5	16-hydroxyhexadecanoic acid	HYDRA	13
6	chenodeoxycholic acid/isomer	CHEA	12
7	chebulic acid	CHE	12
8	gluconic acid	GLUA	11
9	corilagin	COR	11
10	ononin	ONO	10
11	erucamide	ERU	10
12	hamamelitannin	HAM	10
13	glycoursodeoxycholic acid	GLYCA	9
14	glycocholic acid	GLYCH	9
15	gallic acid	GAL	9
16	glycyrrhizic acid	GLYCZ	8
17	glycyrrhetinic acid	GLYCT	8
18	glycycoumarin	GLYC	8

19	hyodeoxycholic acid	HYO	6
20	hexadecanoic acid	HEXA	6
21	linoleic acid	LIN	5
22	licoricesaponin G2	LIC	5
23	isoliquiritin	ISO	5
24	indirubin	IND	5
25	liquiritin	LIQ	4
26	liquiritigenin	LIQN	4
27	linolenic acid	LINA	4
28	oleic acid	OLEA	3
29	oleamide	OLE	3
30	octaethylene glycol	OCT	3
31	tauroursodeoxycholic acid	TAUD	2
32	taurocholic acid	TAUC	2
33	piperine	PIP	2

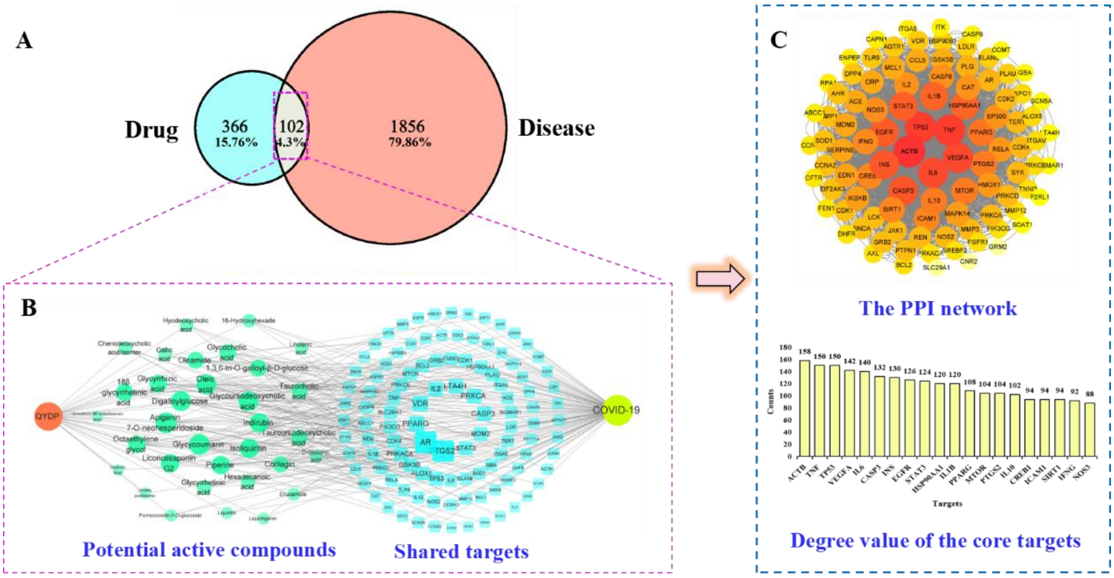

Figure S1 Network pharmacology analysis. Venn diagram of the shared targets of QDP and COVID-19 **(A)**. The network of preparation-potential active compounds-shared targets-disease. (The green nodes represented the active compounds and the blue nodes were the shared targets, and the node size was ranked according to the degree value.) **(B)**. The PPI network and degree value of core targets **(C)**.

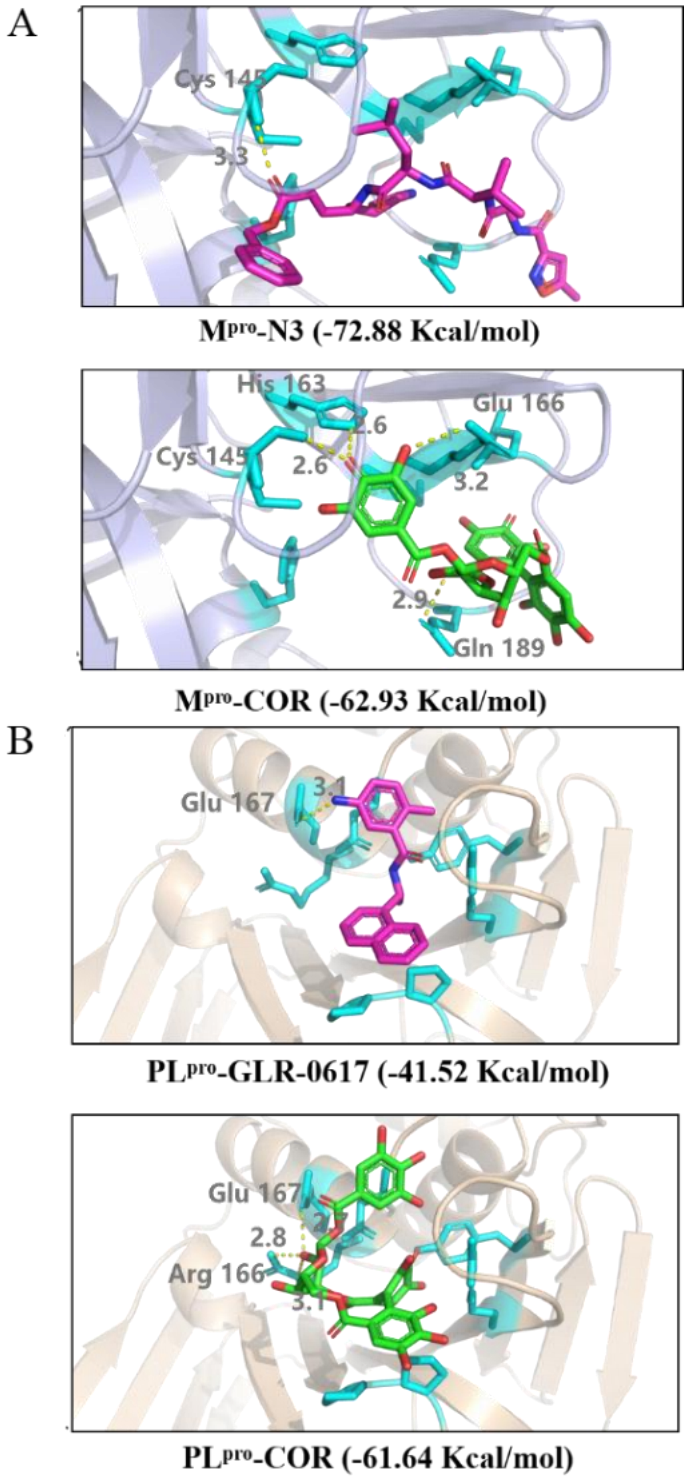

Figure S2 Molecular docking of M^{pro}-N3 and M^{pro}-COR **(A),** and PL^{pro}-GRL0617 and PL^{pro}-COR **(B)**.

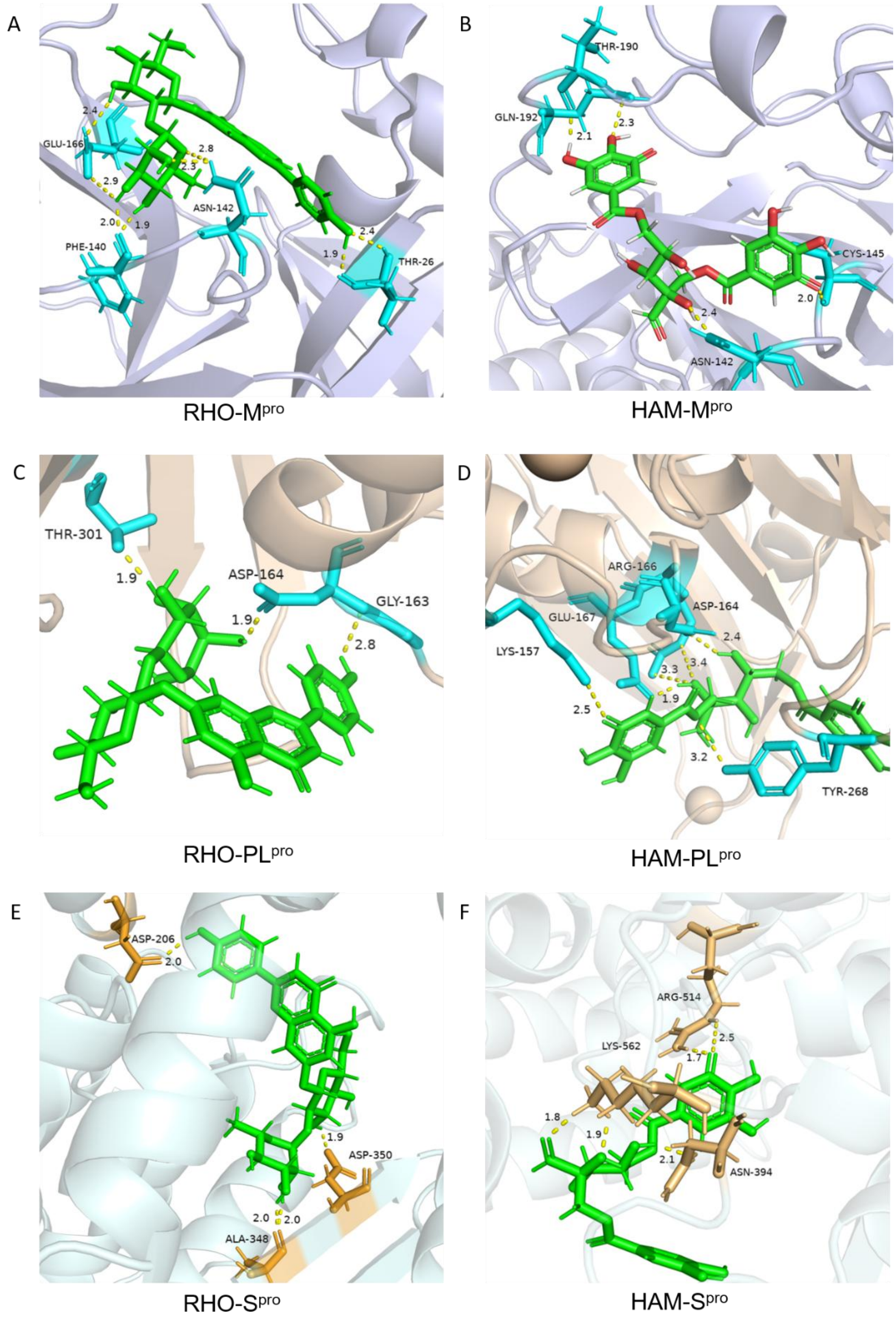

Figure S3 Binding conformations of RHO-M^{pro} complex (**A**). Binding conformations of HAM-M^{pro} complex (**B**). Binding conformations of RHO-PL^{pro} complex (**C**). Binding conformations of HAM-PL^{pro} complex (**D**). Binding conformations of RHO-S^{pro} complex (**E**). Binding conformations of RHO-S^{pro} complex (**F**).

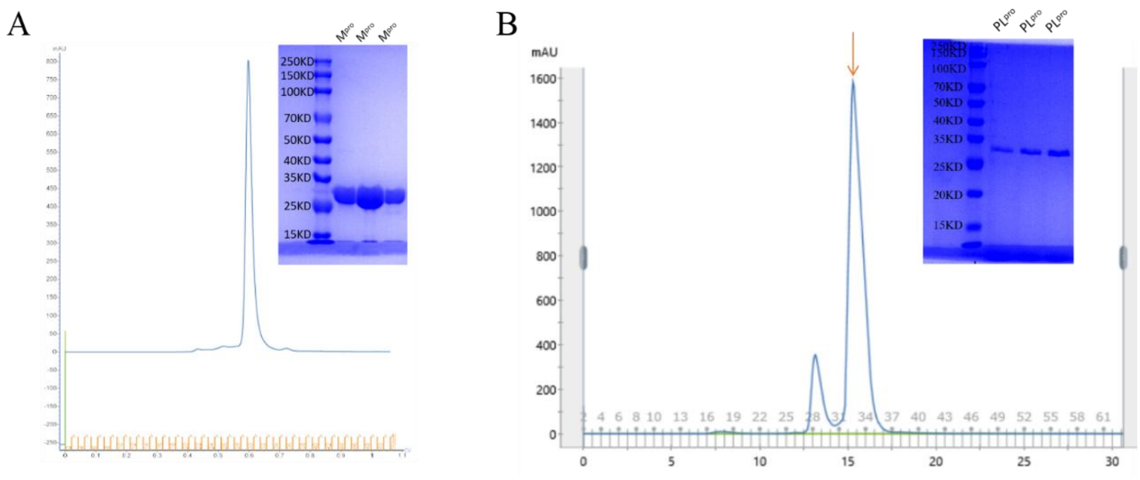

Figure S4 The purification of M^{pro} (A) and PL^{pro} (B).

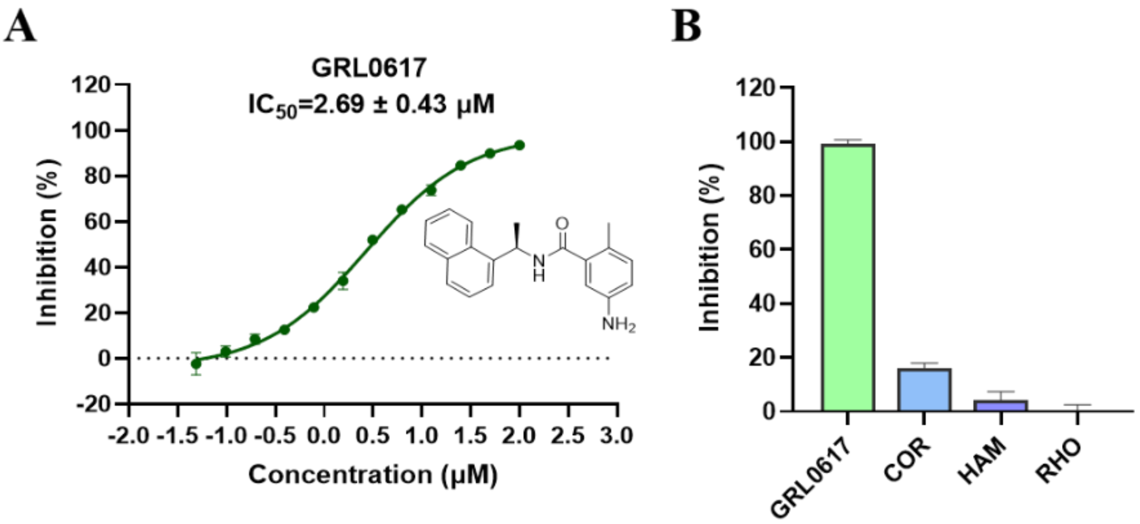

Figure S5 Dose–response curves for IC$_{50}$ values of GLR0617 (A). Compounds lead to inhibit the activity of SARS-CoV-2 PL^{pro} (B) at 40 μM. All data are shown as mean ± s.e.m. (n = 3)

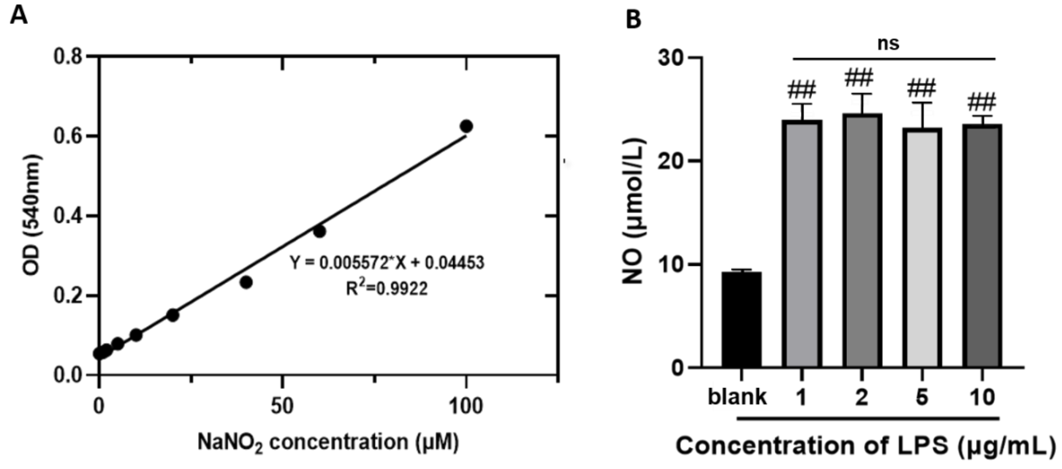

Figure S6 Standard curve for determination of NO concentration (A); Determination of the LPS concentration (B). # indicates vs the blank group.

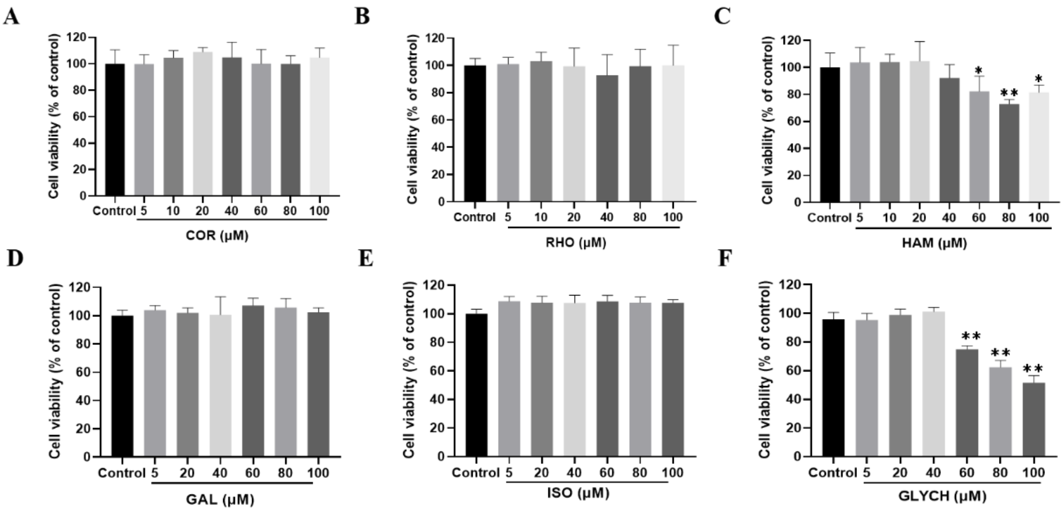

Figure S7 Influence on cell viability of RAW 264.7 cells by COR (A), RHO (B), HAM (C), GAL (D), ISO (E), and GLYCH (F).

Reference

1.Pfundstein B, El Desouky SK, Hull WE, Haubner R, Erben G, Owen RW. Polyphenolic compounds in the fruits of Egyptian medicinal plants (Terminalia bellerica, Terminalia chebula and Terminalia horrida): characterization, quantitation and determination of antioxidant capacities. *Phytochemistry*. (2010) 71(10): 1132-48. doi.org/10.1016/j.phytochem.2010.03.018

2. Kumar S, Chandra P, Bajpai V, Singh A, Srivastava M, Mishra DK, et al. Rapid qualitative and quantitative analysis of bioactive compounds from Phyllanthus amarus using LC/MS/MS techniques. *Industrial Crops & Products*. (2015) 69:143-152. doi.org/10.1016/j.indcrop.2015.03.023

3. Zhang X, Liang C, Yin J, Sun Y, Zhang L. Identification of metabolites of liquiritin in rats by UHPLC-Q-TOF-MS/MS: metabolic profiling and pathway comparison in vitro and in vivo. *RSC Advances*. (2018) 8(21): 11813-11827. doi.org/10.1039/C8RA00583F

4. Du Y, Li C, Xu S, Yang J, Wan H, He Y. LC-MS/MS combined with blood-brain dual channel microdialysis for simultaneous determination of active components of astragali radix-safflower combination and neurotransmitters in rats with cerebral ischemia reperfusion injury: Application in pharmacokinetic and pharmacodynamic study. *Phytomedicine*. (2022) 106:154432-154447. doi.org/10.1016/j.phymed.2022.154432

5. Yan Z, Chen Y, Li T, Zhang J, Yang X. Identification of metabolites of Si-Ni-San, a traditional Chinese medicine formula, in rat plasma and urine using liquid chromatography/diode array detection/triple-quadrupole spectrometry. *Journal of Chromatography B: Analytical Technologies in the Biomedical and Life Sciences*. (2012) 885-886:73-82. doi.org/10.1016/j.jchromb.2012.04.030

6. Chen D, Lin S, Xu W, Huang M, Chu J, Xiao F, et al. Qualitative and quantitative analysis of the major constituents in Shexiang Tongxin dropping pill by HPLC-Q-TOF-MS/MS and UPLC-QqQ-MS/MS. *Molecules*. (2015) 20(10): 18597-18619. doi.org/10.3390/molecules201118597

7. Jia X, Liu Y, Wang S, Ma J, Yu J, Yue X, et al. Screening of metabolic markers present in Oxytropis by UHPLC-Q-TOF/MS and preliminary pharmacophylogenetic investigation. *Frontiers in Plant Science*. (2022) 13:958460-958476. doi.org/10.3389/fpls.2022.958460

8.Yan T, Fu Q, Wang J, Ma S. UPLC-MS/MS determination of ephedrine, methylephedrine, amygdalin and glycyrrhizic acid in Beagle plasma and its application to a pharmacokinetic study after oral administration of Ma Huang Tang. *Drug Testing and Analysis*. (2015) 7(2):158-163. doi.org/10.1002/dta.1680

9. Liu Y, Tan P, Liu S, Shi H, Feng X, Ma Q. A new method for identification of natural, artificial and in vitro cultured Calculus bovis using high-performance liquid chromatography-mass spectrometry. *Pharmacognosy Magazine*. (2015) 11(42): 304-310. doi.org/10.4103/0973-1296.156575

10. Li YJ, Chen J, Li Y, Li Q, Zheng YF, Fu Y, et al. Screening and characterization of natural antioxidants in four Glycyrrhiza species by liquid chromatography coupled with electrospray ionization quadrupole time-of-flight tandem mass spectrometry. *Journal of Chromatography A*. (2011) 1218(45): 8181-8191. doi.org/10.1016/j.chroma.2011.07.073

11. Hu J, Chang H, Wang L, Wu S, Shao B, Zhou J, et al. Detection, occurrence and fate of indirubin in municipal sewage treatment plants. *Environmental Science & Technology*. (2008) 42(22): 8339-8344. doi.org/10.1021/es801105w

12. Shan L, Yang N, Zhao Y, Sheng X, Yang S, Li Y. A rapid classification and identification method applied to the analysis of glycosides in Bupleuri radix and liquorice by ultra high performance liquid chromatography coupled with quadrupole time-of-flight mass spectrometry. *Journal of Separation Science*. (2018) 41(19): 3791-3805. doi.org/10.1002/jssc.201800406

13. Naik DG, Puntambekar H, Anantpure P. Essential oil of Terminalia chebula fruits as a repellent for the Indian honeybee Apis florea. *Chemical Biodiversity*. (2010) 7(5): 1303-1310. doi.org/10.1002/cbdv.201000035
